# Supplementary material for: Synergistic dual-phase air electrode enables high and durable performance of reversible proton ceramic electrochemical cells
Source: Nat Commun. 2024 Jan 11;15:472. doi: 10.1038/s41467-024-44767-5 (PMC10784466; doi:10.1038/s41467-024-44767-5)
Supplement: Supplementary file 1 — Supplementary Information [file 41467_2024_44767_MOESM1_ESM.pdf]

Supplementary Information

Synergistic dual-phase air electrode enables high and durable performance of reversible proton ceramic electrochemical cells

Zuoqing Liu[1], Yuesheng Bai[1], Hainan Sun[2], Daqin Guan[3], Wenhuai Li[1], Wei-Hsiang Huang[4], Chih-Wen Pao[4], Zhiwei Hu[5], Guangming Yang[1], Yinlong Zhu[6*], Ran Ran[1], Wei Zhou[1], Zongping Shao[1,7*]*

[1] State Key Laboratory of Materials-Oriented Chemical Engineering, College of Chemical Engineering, Nanjing Tech University, Nanjing, 211816, PR China

[2] Department of Materials Science and Engineering, Korea Advanced Institute of Science and Technology (KAIST), Daejeon, 34141, Republic of Korea

[3] Department of Building and Real Estate, Research Institute for Sustainable Urban Development (RISUD) and Research Institute for Smart Energy (RISE), The Hong Kong Polytechnic University, Kowloon, China

[4] National Synchrotron Radiation Research Center, 101 Hsin-Ann Road, Hsinchu 30076, Taiwan

[5] Max-Planck-Institute for Chemical Physics of Solids, Nöthnitzer Str. 40, Dresden 01187, Germany

[6] Institute for Frontier Science, Nanjing University of Aeronautics and Astronautics, Nanjing 210016, PR China

[7] WA School of Mines: Minerals, Energy and Chemical Engineering (WASM-MECE), Curtin University, Perth, WA 6845, Australia

* Corresponding authors:

Guangming Yang: ygm89525@njtech.edu.cn

Yinlong Zhu: zhuyl1989@nuaa.edu.cn

Zongping Shao: shaozp@njtech.edu.cn

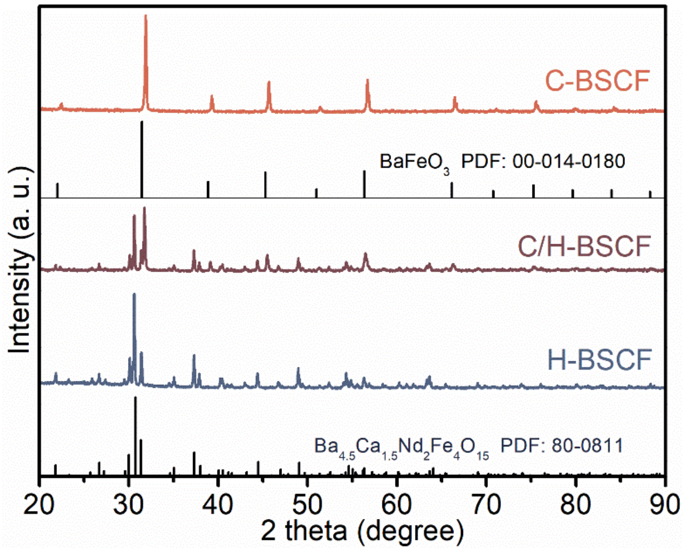

Supplementary Figure 1. XRD patterns of C-BSCF, C/H-BSCF and H-BSCF.

The X-ray diffraction (XRD) patterns of $Ba_{0.5}Sr_{0.5}Co_{0.8}Fe_{0.2}O_{3-\delta}$ (C-BSCF), $Ba_{1.5}Sr_{1.5}Co_{1.6}Fe_{0.4}O_{7-\delta}$ (C/H-BSCF) and $Ba_4Sr_4Co_{3.2}Fe_{0.8}O_{16-\delta}$ (H-BSCF) powders after calcination at 1000 °C for 5 h in still air are shown in Supplementary Fig. 1. All observed peaks of the as-prepared C-BSCF oxide led to the cubic perovskite structure, while H-BSCF exhibits the characteristic peaks of hexagonal perovskite without any other impurity peaks present. Meanwhile, the diffraction peaks of the synthesized C/H-BSCF coincide with the superposition of C-BSCF and H-BSCF peaks, so it is tentatively concluded that C/H-BSCF is composed of cubic C-BSCF and hexagonal H-BSCF phases together.

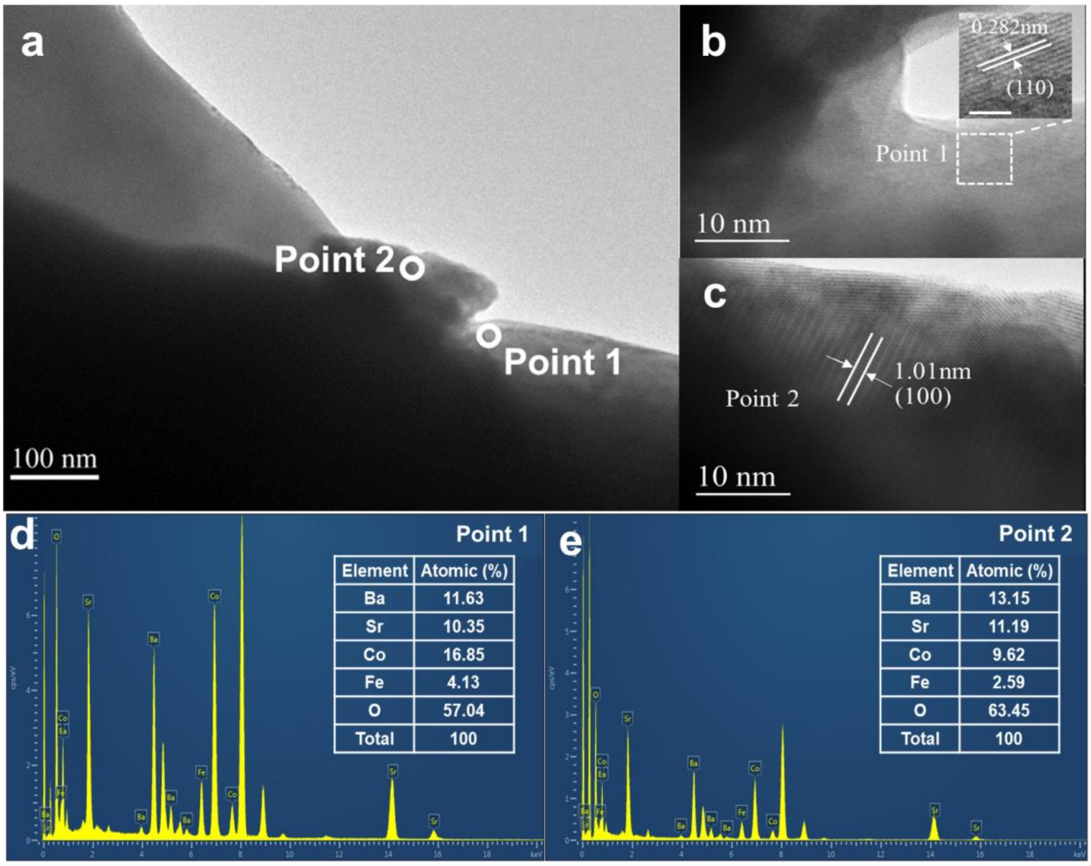

Point 1

Element	Atomic (%)
Ba	11.63
Sr	10.35
Co	16.85
Fe	4.13
O	57.04
Total	100

Point 2

Element	Atomic (%)
Ba	13.15
Sr	11.19
Co	9.62
Fe	2.59
O	63.45
Total	100

Supplementary Figure 2. Phase composition and crystal structure analysis of the hybrid C/H-BSCF. **a** STEM image of C/H-BSCF particle. HR-TEM images of the (**b**) point 1 and (**c**) point 2 regions, respectively. The inset shows localized enlargement (The scale bar in the inset is 2 nm). Point EDX scanning results at (**d**) point 1 and (**e**) point 2. The inserted tables in the Supplementary Fig. 2d, e show the atomic ratios of the elements.

Supplementary Fig. 2a presents the scanning transmission electron microscopy (STEM) image of the C/H-BSCF powder. To further investigate the phase structure and composition of the hybrid C/H-BSCF, we conducted high-resolution transmission electron microscopy (HR-TEM). At points 1 and 2, diffraction patterns corresponding to the (110) and (100) planes of C-BSCF and H-BSCF phases were detected, revealing lattice spacings of 0.282 nm and 1.01 nm, respectively (Supplementary Fig. 2b, c). EDX scanning at points 1 and 2 indicated the presence of Ba, Sr, Co, Fe, and O in both phases, with the main difference lying in the ratios of the A-site elements Ba and Sr to the B-site elements Co and Fe (Supplementary Fig. 2d, e). Point EDX scanning results show that the elemental compositions of the cubic and hexagonal phases in C/H-BSCF are in general agreement with the expected design composition.

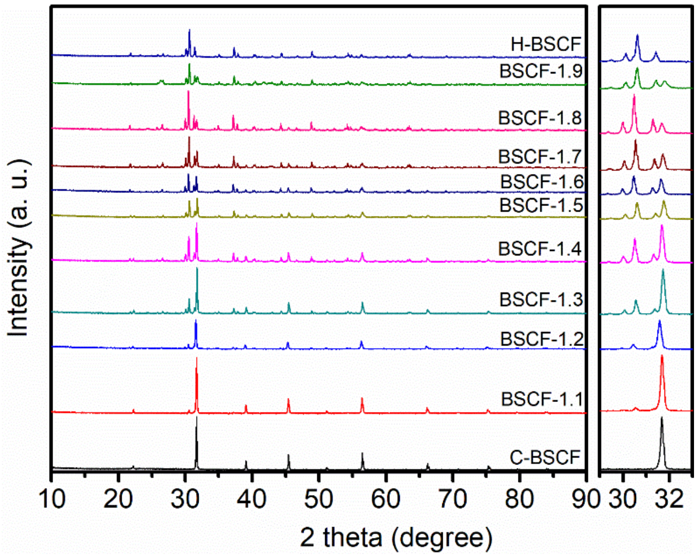

Supplementary Figure 3. XRD patterns of $(Ba_{0.5}Sr_{0.5})_xCo_{0.8}Fe_{0.2}O_{2+x-\delta}$ oxides (BSCF-*x*, where *x* varies from 1 to 2 in increments of 0.1).

To investigate the applicability of the strategy of modulating the content of alkaline earth metals at the A-site to induce the formation of dual-phase hybrids, we synthesized a number of air electrodes $(Ba_{0.5}Sr_{0.5})_xCo_{0.8}Fe_{0.2}O_{2+x-\delta}$ (BSCF-*x*, where *x* varies from 1 to 2 in increments of 0.1). The XRD patterns of the samples are shown in the Supplementary Fig. 3, which clearly reveals that C-BSCF and H-BSCF are single cubic and hexagonal perovskite phases, respectively, while the rest of the samples BSCF-*x* (*x* = 1.1, 1.2, 1.3, 1.4, 1.5, 1.6, 1.7, 1.8, and 1.9) show characteristic peaks of composites composed of cubic and hexagonal phases. In addition, it is observed from the peak intensities that the hexagonal phase content increases with the increase of the alkaline earth metal content at the A-site[1].

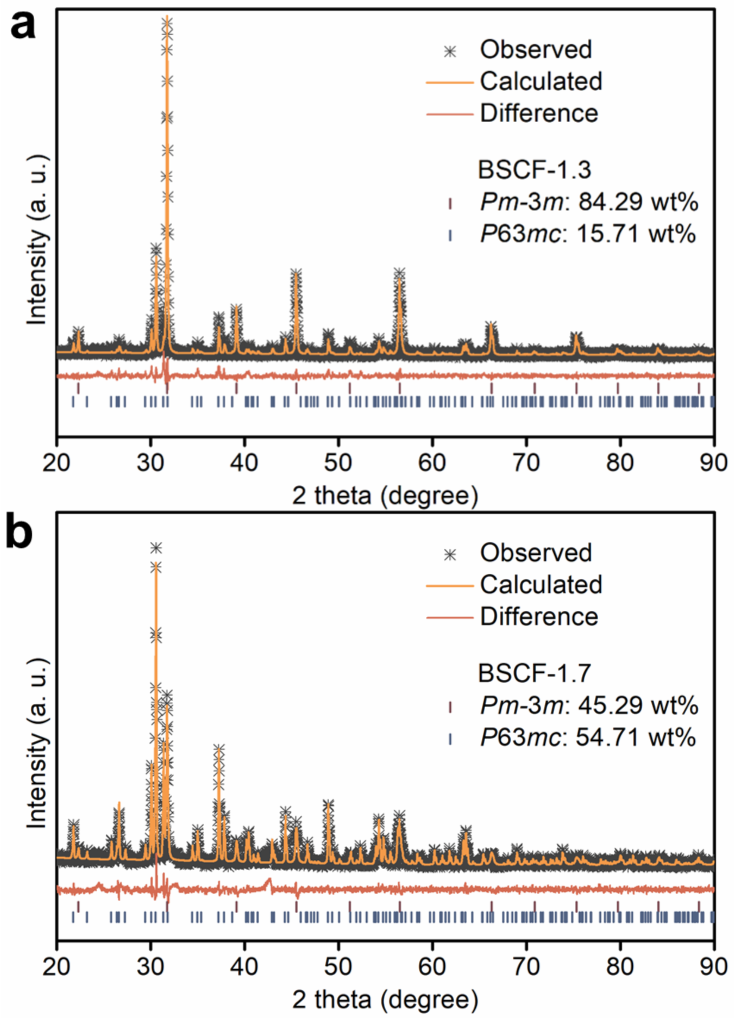

Supplementary Figure 4. Refined XRD profiles of the prepared (**a**) BSCF-1.3 and (**b**) BSCF-1.7 oxides.

To determine the A-site modulation is capable of changing the ratio of the two phases in the hybrids[2], Rietveld refinement of the XRD data of the BSCF-1.3 and BSCF-1.7 samples was performed with the results shown in Supplementary Fig. 4a, b. The content of the cubic and hexagonal phases in the BSCF-1.3 sample was 84.29 wt% and 15.71 wt%, respectively. The cubic phase of the hybrid BSCF-1.3 has a space group of *Pm-3m* with lattice parameters of a = b = c = 3.9917(0) Å, and the hexagonal phase has a space group of *P63mc* with lattice parameters of a = b = 11.7077(2) Å and c = 6.9066(3) Å (R_{exp} = 4.90%, R_{wp} = 6.84%, *GOF* = 1.40). In addition, BSCF-1.7 samples contained 45.29 wt% and 54.71 wt% of cubic and hexagonal phases, respectively, and the lattice parameters of the two phases are shown in Supplementary Table 1.

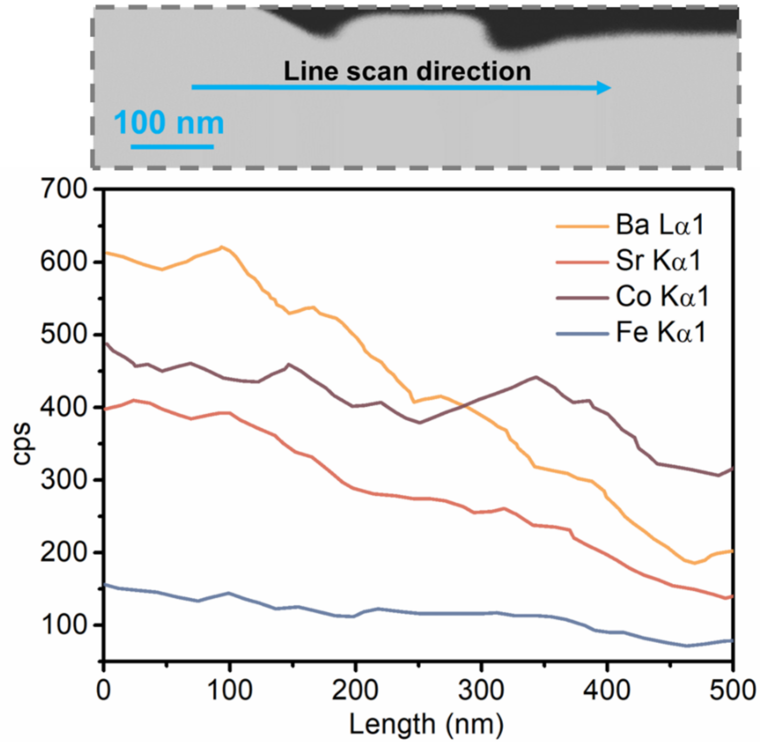

Supplementary Figure 5. Energy dispersive X-ray (EDX) spectroscopy line-scan profiles of C/H-BSCF oxide.

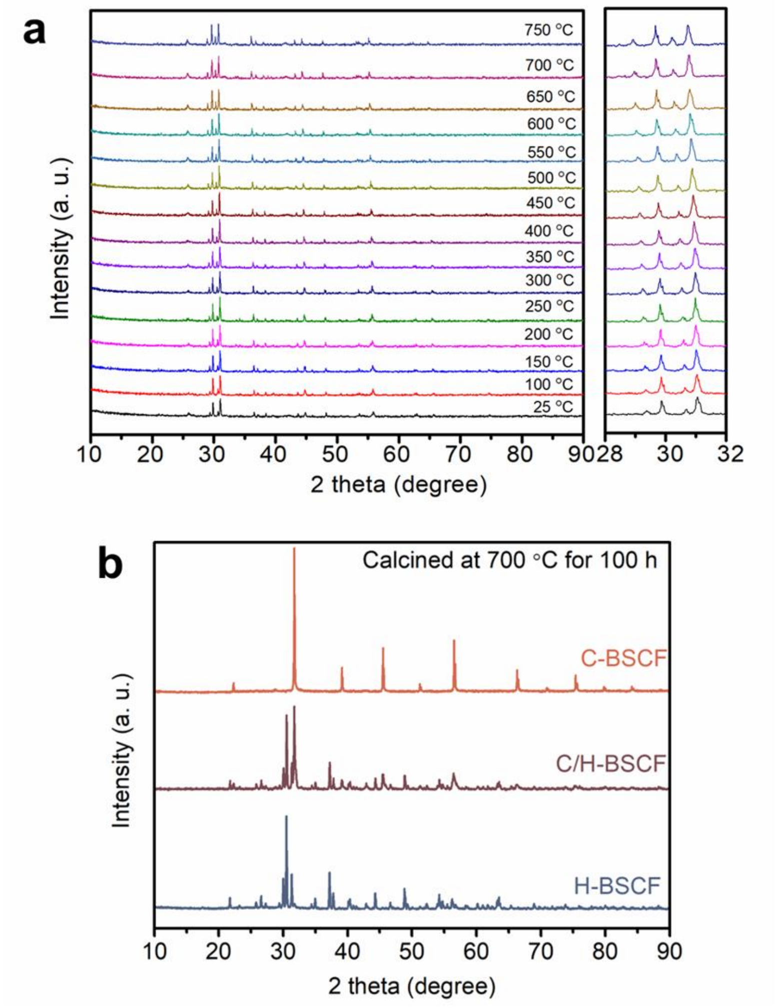

Supplementary Figure 6. a High-temperature XRD patterns of C/H-BSCF. **b** XRD patterns of C-BSCF, C/H-BSCF and H-BSCF powders after calcination at 700 °C for 100h.

Through high temperature *in-situ* XRD, it can be clearly found that the C/H-BSCF powder still maintains a stable crystal structure in the temperature range from room temperature to 750 °C (Supplementary Fig. 6a). Meanwhile, the hybrid C/H-BSCF still shows the characteristic peaks of cubic and hexagonal phases without impurity peaks. With increasing temperature, reasonable lattice expansion was also found due to the shift of the characteristic peaks to lower degree[3]. Supplementary Fig. 6b exhibits the XRD patterns of C-BSCF, C/H-BSCF and H-BSCF powders after calcination at 700 °C for 100 h. It is observed that the three samples maintain their original phase structure and composition after long calcination, indicating that C-BSCF, C/H-BSCF and H-BSCF powders have excellent thermal stability.

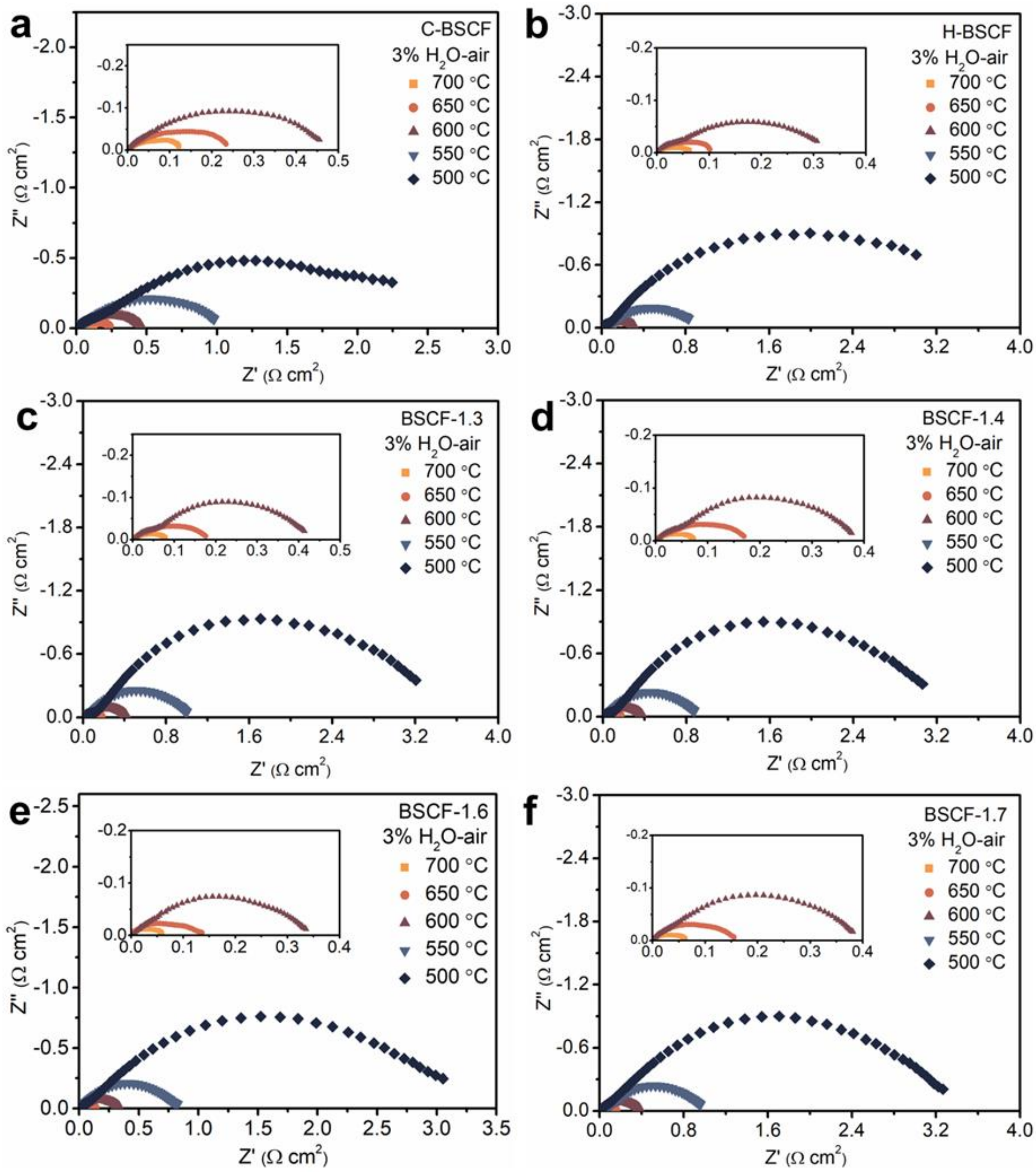

Supplementary Figure 7. a-f EIS plots of BZCYYb-supported symmetric cells with C-BSCF, H-BSCF and BSCF-*x* electrodes (*x* = 1.3, 1.4, 1.6, and 1.7) at 500-700 °C under 3% H_2O-air.

The oxygen activation performance of the air electrode was determined from the area specific resistance (ASR), which was obtained via electrochemical impedance spectroscopy (EIS) in a symmetrical cell (electrode|BZCYYb|electrode). The typical EIS of the symmetric cell with C-BSCF air electrode in 3% H_2O-air with ASRs of 0.126, 0.243, 0.478, 1.093, and 3.390 Ω cm^2 at 700, 650, 600, 550, and 500 °C, respectively. Under the same test conditions, the ASRs of the symmetric cell with H-BSCF electrode are 0.063, 0.117, 0.343, 0.979, and 4.002 Ω cm^2 at 700, 650, 600, 550, and 500 °C, respectively. compared with the C-BSCF electrode, H-BSCF has better oxygen activation at higher temperatures, while the activity decays with decreasing temperature faster. Thus, owing to the synergistic effect of the dual phases, the hybrid air electrode has higher electrochemical activity than the C-BSCF at high temperatures, while the

hybrid electrode outperforms the H-BSCF electrode at lower test temperatures. For instance, the cell with BSCF-1.4 electrode has the ASRs of 0.074, 0.171, 0.387, 0.909, and 3.245 Ω cm^2 at 700, 650, 600, 550, and 500 °C, respectively.

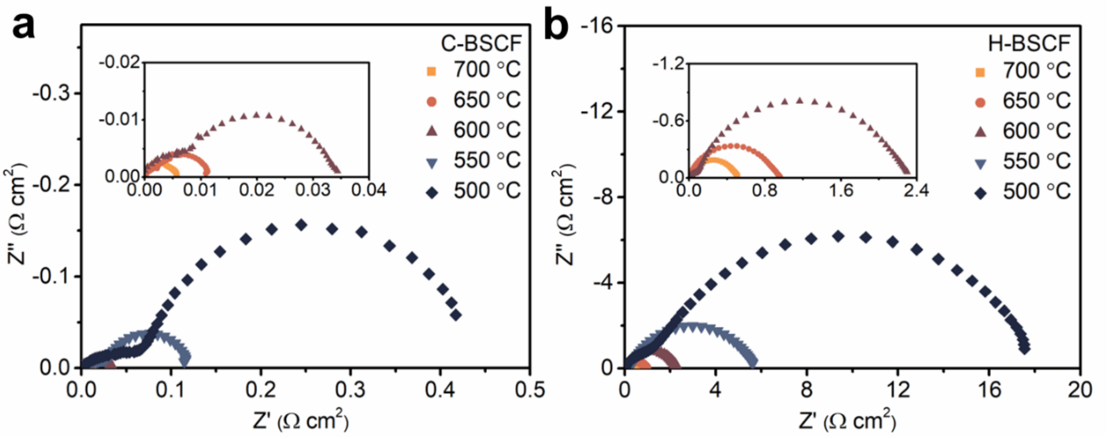

Supplementary Figure 8. EIS of (**a**) C-BSCF and (**b**) H-BSCF electrodes on symmetric cells using SDC electrolyte at 500-700 ℃ under dry air.

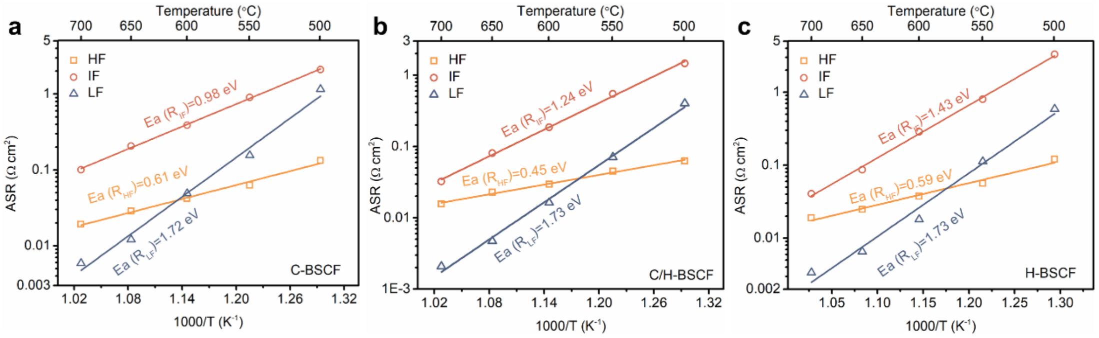

Supplementary Figure 9. Temperature dependence of ASRs of the (**a**) C-BSCF, (**b**) C/H-BSCF and (**c**) H-BSCF electrodes under wet air (3% H_2O).

Supplementary Fig. 9 shows the temperature dependence of the ASR of C-BSCF, C/H-BSCF and H-BSCF electrodes in HF, IF, and LF regions. For the three different electrodes the ASR in the IF region remains predominant in the tested temperature range of 700-500 °C. The activation energy of the C/H-BSCF electrode is 1.24 eV, which is in the middle of the activation energies of C-BSCF and H-BSCF. This is mostly due to the different rates of ionic conduction promotion between the cubic and hexagonal phases in the C/H-BSCF hybrid electrode. The impedance increase is most pronounced in the LF region with decreasing temperature, and the activation energies of the C-BSCF, C/H-BSCF and H-BSCF electrodes in the LF region are all around 1.73 eV. In the HF region, the C/H-BSCF electrode has the lowest activation energy of 0.45 eV, indicating a fast charge transfer and oxygen activation rate at the three-phase boundary (TPB) compared to the single-phase C-BSCF and H-BSCF.

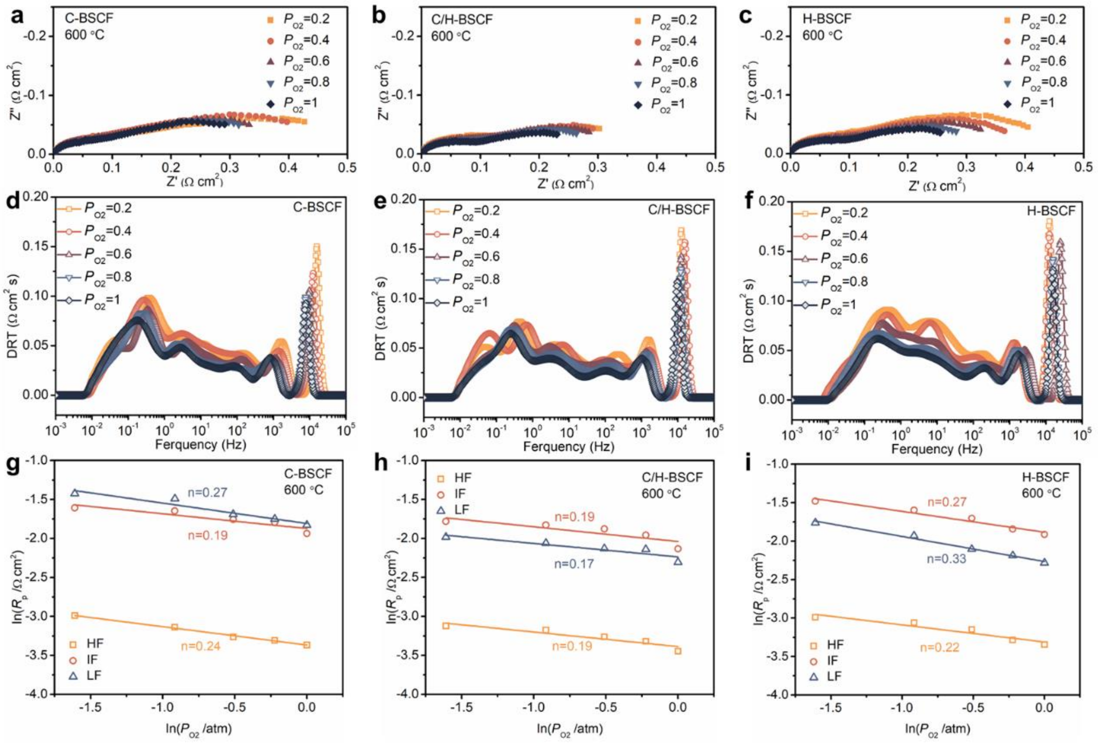

Supplementary Figure 10. a, b, c EIS and (**d, e, f**) DRT plots of C-BSCF, C/H-BSCF, and H-BSCF electrodes as a function of oxygen partial pressure. **g, h, i** Dependence of R_p on oxygen partial pressure for C-BSCF, C/H-BSCF, and H-BSCF electrodes in the HF, IF and LF regions.

As shown in Supplementary Fig. 10 a,b,c, the ORR kinetic of the C-BSCF, C/H-BSCF, and H-BSCF air electrodes were investigated by measuring the EIS at 600 °C under different P_{O2}. When the air electrode was exposed to pure oxygen, as expected, the symmetric cell demonstrated a relatively low R_p. As the P_{O2} decreased, the R_p showed a significant increase. Shown in Supplementary Fig. 10 d,e,f, are the DRT plots of the electrochemical processes for C-BSCF, C/H-BSCF, and H-BSCF at different P_{O2} at 600 °C. The integral area of each process corresponds to the polarization resistance of each process, and the general relationship between R_p and P_{O2} follows the equation $R_p = k(P_{O2})^{-n}$. The C/H-BSCF hybrid electrode exhibits the lowest R_p at different oxygen partial pressures at 600 °C. However, in the HF region, the C-BSCF, C/H-BSCF, and H-BSCF electrodes show similar R_p and n values of about 0.2, indicating that the charge transfer processes of different electrodes do not significantly discrepancy. The lower R_p of C/H-BSCF electrode is mainly attributed to the decrease in resistance of the IF and LF regions compared to C-BSCF and H-BSCF electrodes. As shown in Supplementary Fig. 10 g,h,i, C-BSCF and H-BSCF electrodes exhibit lower R_p in the IF and LF regions, respectively, indicating that the rapid ORR rate of the C/H-BSCF hybrid electrode is mainly attributed to the promotion of oxygen ion diffusion rate and surface mass transfer by the cubic and hexagonal phases, respectively.

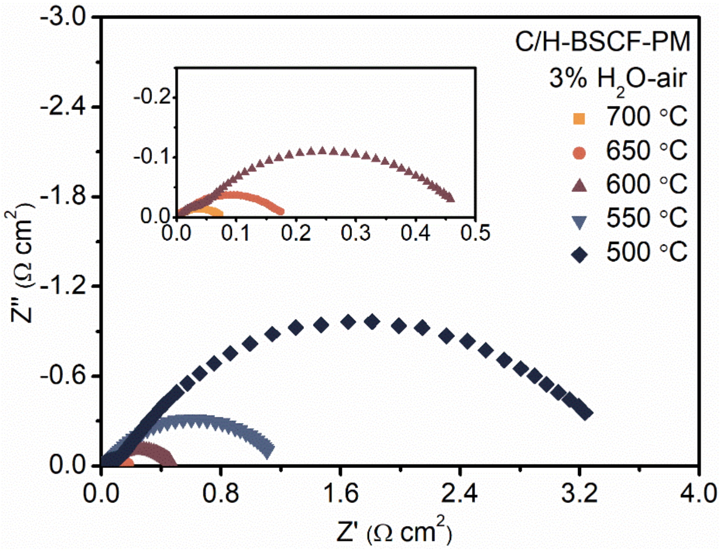

Supplementary Figure 11. EIS plots of symmetric cell with C/H-BSCF-PM electrode at 500-700 °C under 3% H_2O-air.

To gain insight into the dual-phase synergistic effect of the hybrid electrode C/H-BSCF on the ORR activity, we supplementally tested the EIS of the C/H-BSCF-PM air electrode prepared by physically mixing C-BSCF and H-BSCF powders. as shown in the supplementary Fig. 11, the EIS of this electrode is measured on a symmetric cell with ASRs of 0.08, 0.18, 0.47,1.20, and 3.56 Ω cm^2 at 3% H_2O-air at 700, 650, 600, 550, and 500 °C, respectively.

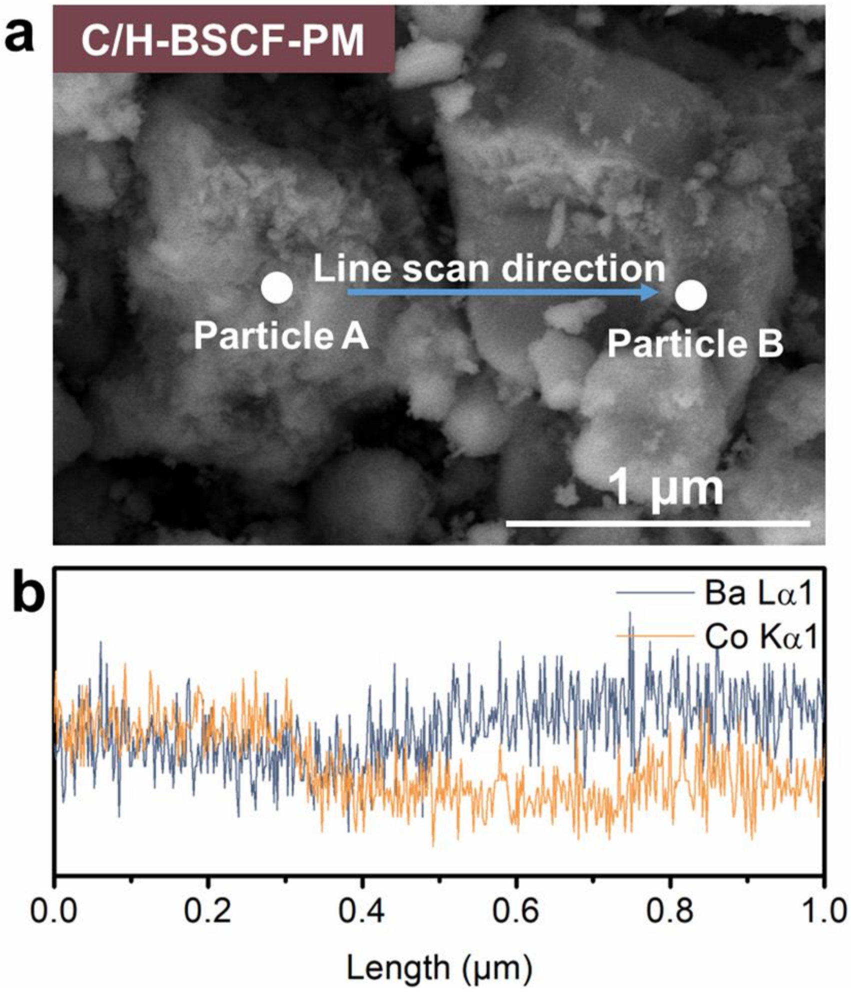

Supplementary Figure 12. a SEM image and (**b**) EDX spectroscopy line-scan profiles of C/H-BSCF-PM oxide.

The particle size of the C/H-BSCF-PM powder is in the micrometer range, as shown in Supplementary Fig. 12a. Since the C-BSCF and H-BSCF phases have the same elemental composition, energy dispersive x-ray (EDX) spectral line scanning was used to detect the A-site Ba element and the B-site Co element, and the difference in the atomic ratios of the A/B sites was utilized to identify the two phases. As seen in Supplementary Fig. 12b, along the direction of the line sweep, the Co content is first higher and then lower, while the Ba content is first lower and then higher, indicating that particles A and B are cubic C-BSCF and hexagonal H-BSCF, respectively. The two phases of the physically mixed C/H-BSCF-PM electrode may be seen to be spread at the micrometer scale. In contrast to the C/H-BSCF-PM electrode, which acts as an ion transport channel through mutual contact between particles, the two phases in C/H-BSCF are distributed in a single particle with a strongly interacting interface between the phases, allowing the air electrode to improve its electrochemical performance by increasing the kinetic rate of the reaction.

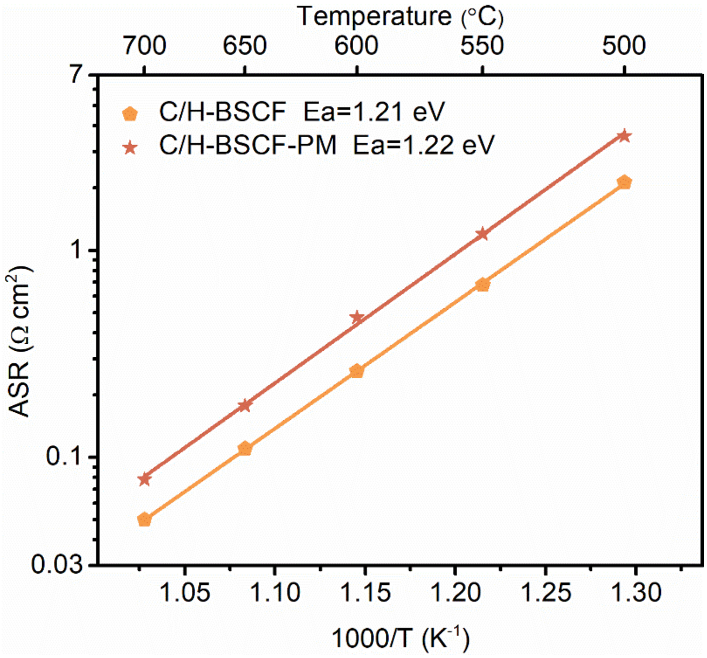

Supplementary Figure 13. Arrhenius plots of the ASRs for C/H-BSCF and C/H-BSCF-PM electrodes.

Supplementary Fig. 13 shows the temperature dependence of ASR for the C/H-BSCF and C/H-BSCF-PM electrodes. the C/H-BSCF and C/H-BSCF-PM electrodes have similar activation energies of 1.21 and 1.22 eV, respectively. The similar temperature dependence of the two electrodes is attributed to the identical phase composition[4]. Furthermore, the C/H-BSCF electrode exhibits a lower ASR at 500-700 °C than the C/H-BSCF-PM electrode. This may be due to the better ion diffusion and surface mass transfer of the self-assembled electrodes compared to the electrodes prepared by physical mixing methods.

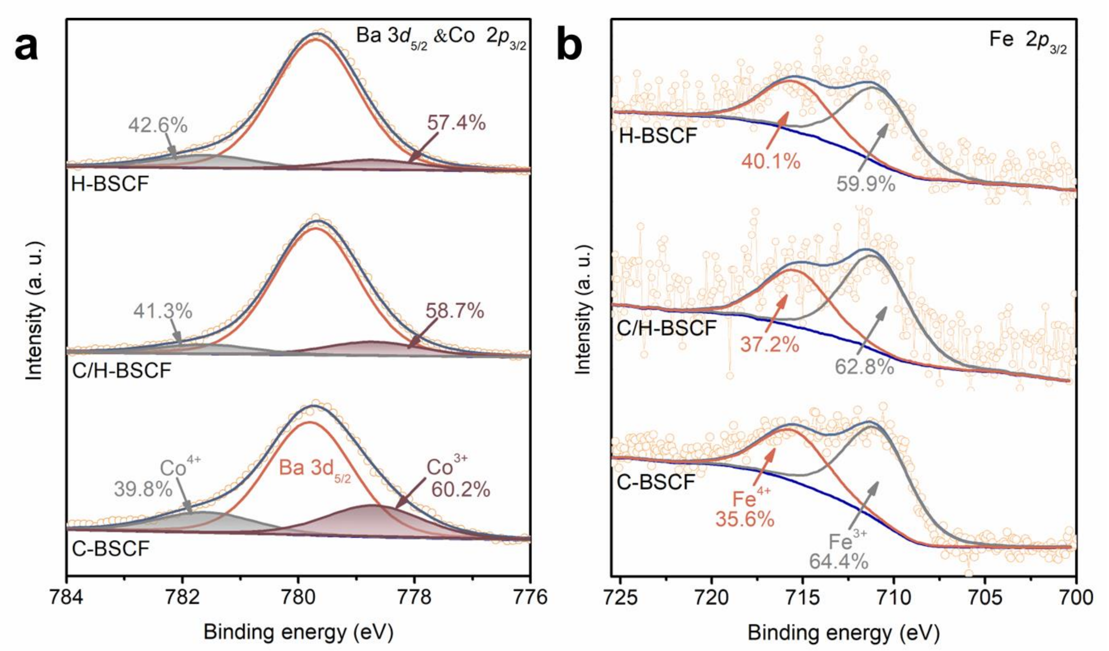

Supplementary Figure 14. The XPS spectra of (**a**) Co 2$p_{3/2}$ and (**b**) Fe 2$p_{3/2}$ for C-BSCF, C/H-BSCF and H-BSCF**.**

In order to understand the initial oxidation states of air electrodes with different phase structures and compositions at room temperature, the valence states of Co and Fe in C-BSCF, C/H-BSCF and H-BSCF electrodes were investigated by X-ray photoelectron spectroscopy (XPS). The XPS fitted plots of Co 2$p_{3/2}$ and Fe 2$p_{3/2}$ in the C-BSCF, C/H-BSCF and H-BSCF samples are exhibited in Supplementary Fig. 14. The binding energies of Co^{3+} and Co^{4+} are 778.5 ± 0.3 and 781.5 ± 0.3 eV, respectively[5]. In the C-BSCF sample, the concentrations of Co^{3+} and Co^{4+} are 60.2% and 39.8%, while they are 57.4% and 42.6% for H-BSCF, respectively. As expected, the concentrations of Co^{3+} and Co^{4+} by the hybrid C/H-BSCF sample were intermediate between the single-phase C-BSCF and H-BSCF. Moreover, the Fe 2$p_{3/2}$ spectra of C-BSCF, C/H-BSCF and H-BSCF were divided into two bands (Fe^{3+} and Fe^{4+})[6]. As displayed in Supplementary Fig. 14b, the valence of Fe in H-BSCF is slightly higher than that in C-BSCF and C/H-BSCF.

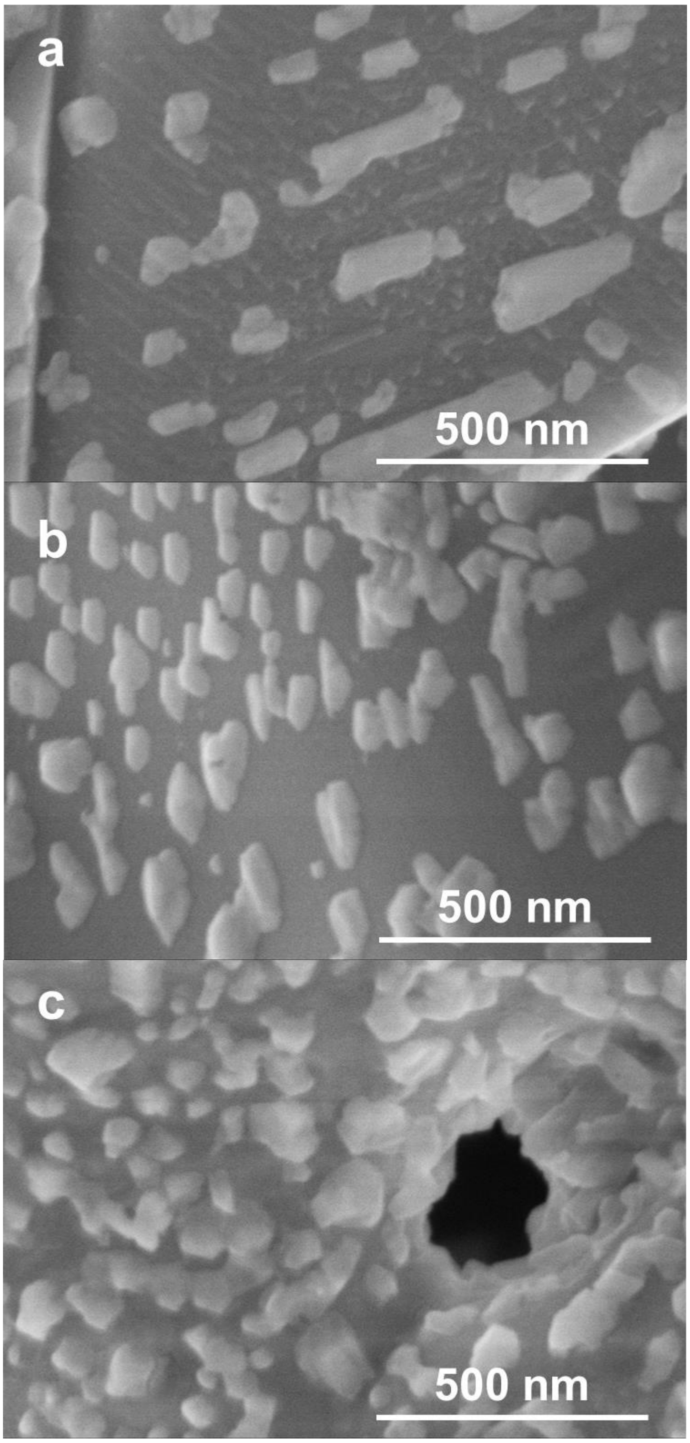

Supplementary Figure 15. the SEM images of (**a**) C-BSCF, (**b**) C/H-BSCF and (**c**) H-BSCF powders.

The surface morphologies of C-BSCF, C/H-BSCF and H-BSCF powders after calcination in ambient air at 1000 °C for 5 h were obtained by SEM. As shown in Supplementary Fig. 15, the surface of H-BSCF samples has more BaO_x particles compared to C-BSCF and C/H-BSCF, which was attributed to the higher content of alkaline earth metals in H-BSCF. In addition, more BaO_x particles on the surface of H-BSCF is beneficial to improve the ORR activity of the air electrode[7].

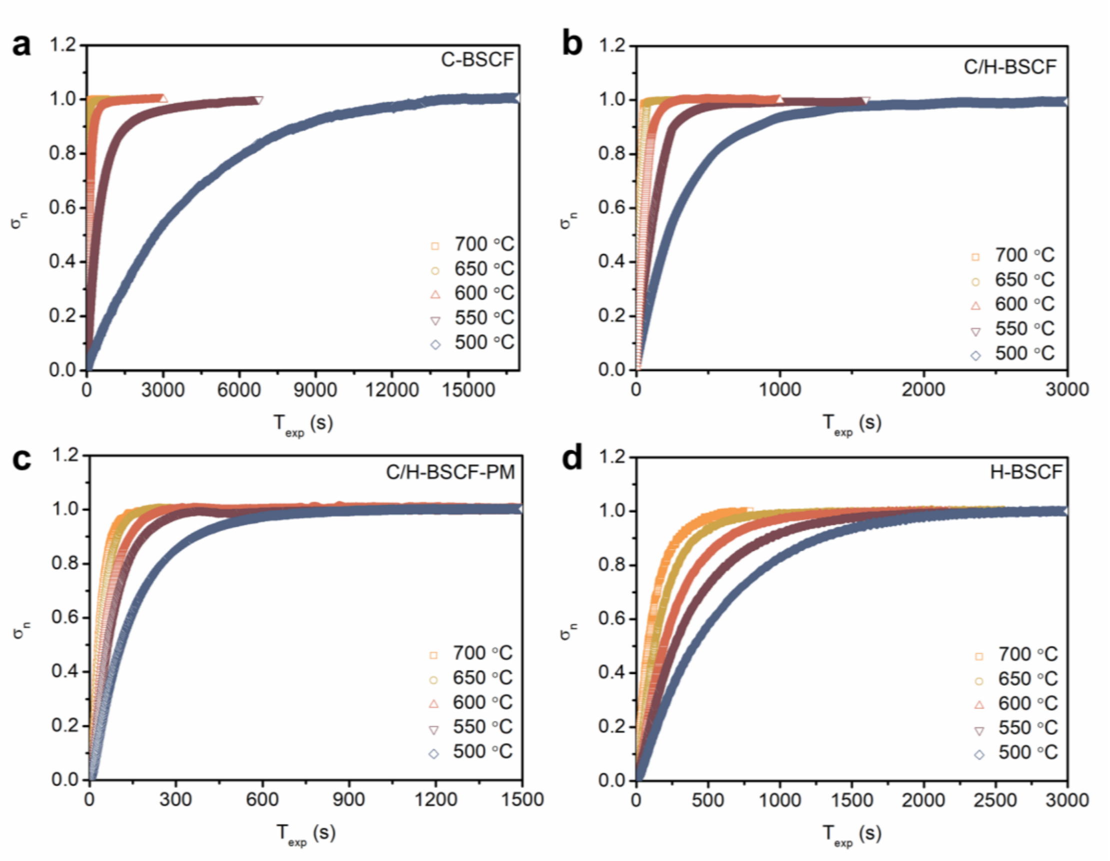

Supplementary Figure 16. ECR curves for (**a**) C-BSCF, (**b**) C/H-BSCF, (**c**) C/H-BSCF-PM and (**d**) H-BSCF samples at 500-700 °C with P_{O2} changes from 21% to 10%. The efficient descriptors (k_{chem} and D_{chem}) for catalytic activity of the air electrode materials were measured by the electrical conductivity relaxation (ECR) technique. Supplementary Fig. 16 shows the normalized conductivity response curves of the C-BSCF and C/H-BSCF samples at 500-700 °C after suddenly changing the P_{O2} from 0.21 to 0.1 atm. The conductivity of C/H-BSCF sample was able to restabilize in a shorter time than C-BSCF sample after oxygen partial pressure conversion, indicating that C/H-BSCF has a faster rate of oxygen surface exchange and bulk phase diffusion.

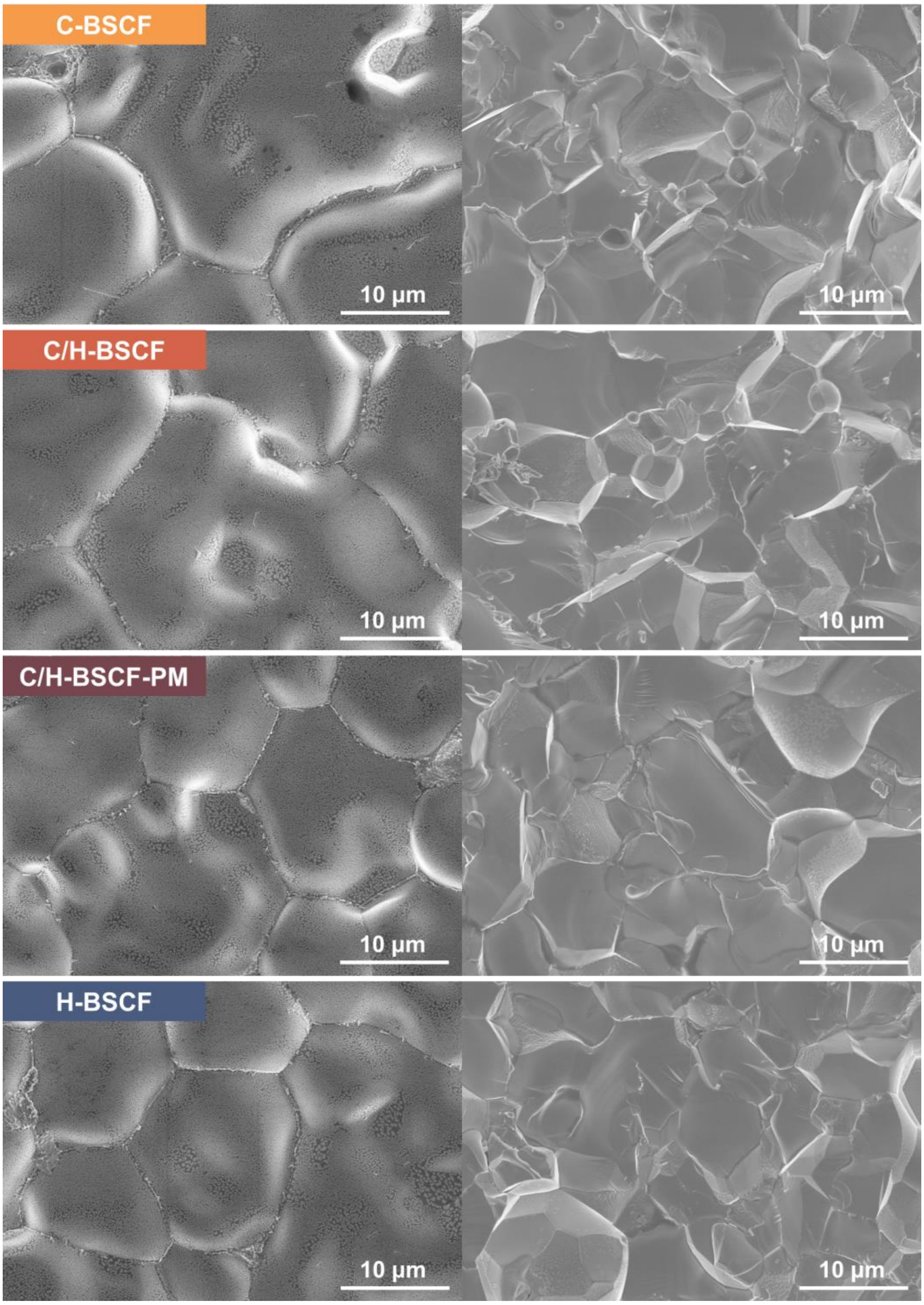

Supplementary Figure 17. SEM images of the surface and cross-section of C-BSCF, C/H-BSCF, C/H-BSCF-PM, and H-BSCF samples.

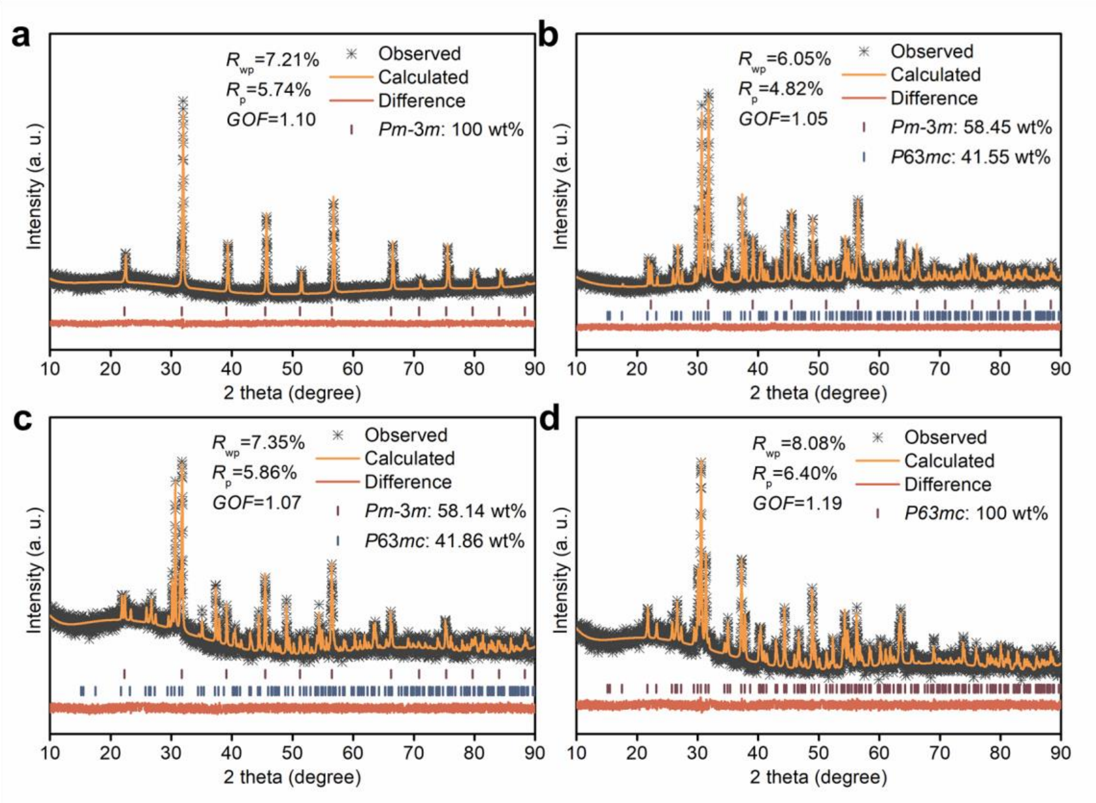

Supplementary Figure 18. Refined XRD profiles of the (**a**) C-BSCF, (**b**) C/H-BSCF, (**c**) C/H-BSCF-PM, and (**d**) H-BSCF bars.

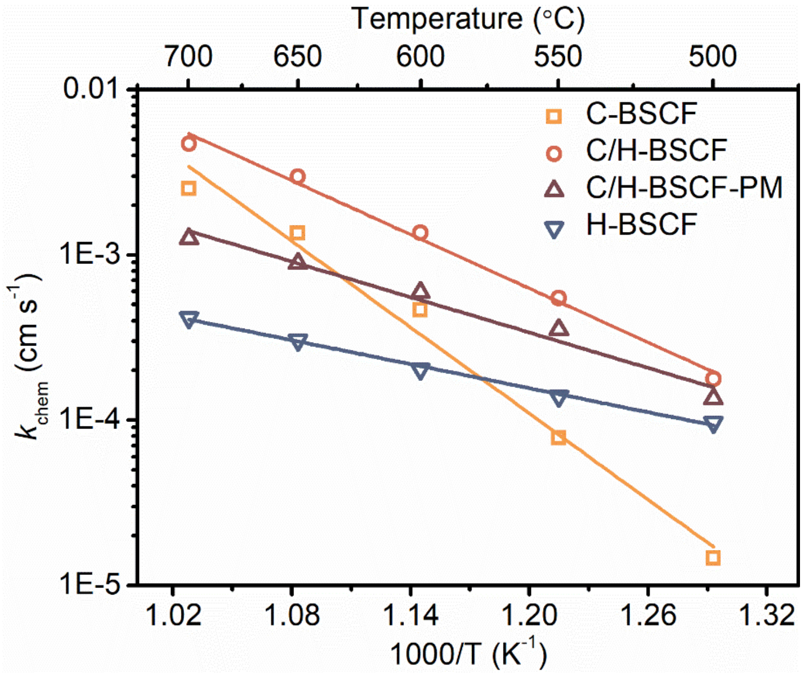

Supplementary Figure 19. Arrhenius plots of the k_{chem} for C-BSCF, C/H-BSCF, C/H-BSCF-PM, and H-BSCF oxides from 500 to 700 °C.

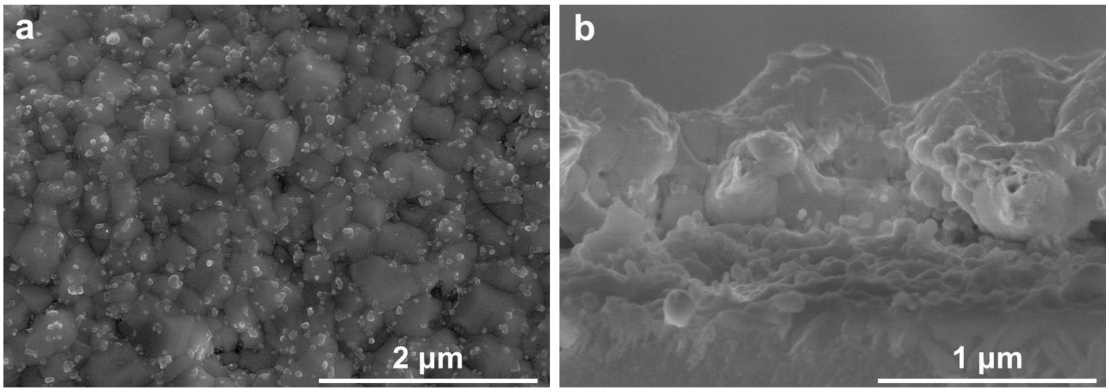

Supplementary Figure 20. SEM images of (**a**) the surface, and (**b**) cross section of the sputtered palladium film.

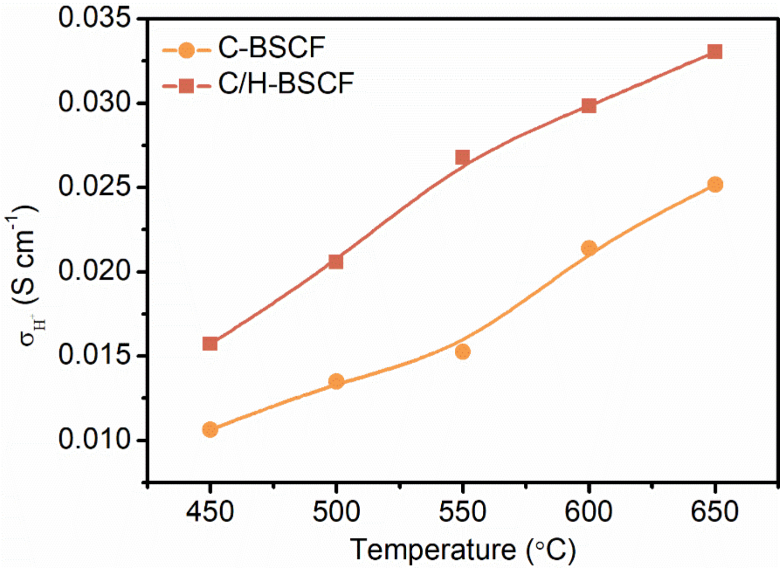

Supplementary Figure 21. Proton conductivities of C-BSCF and C/H-BSCF at 450-650 °C

the proton conductivity of cubic C-BSCF and hybrid C/H-BSCF was computed for quantitative analysis using Eq. 1:

$$\sigma_{H^+} = J_{H2}\frac{4F^2L}{RT} \Big/ \ln\frac{P_{H2,supp.}}{P_{H2,perm.}} \quad (1)$$

Where J_{H2} is H_2 permeation flux (mol cm^{-2} s^{-1}), F is the Faraday constant (96485.3326 C mol^{-1}), L is the membrane thickness, R is the ideal gas constant (8.314 J K^{-1} mol^{-1}), T is the temperature, $P_{H2,\ supp.}$ is H_2 partial pressure at the feed side, and $P_{H2,\ perm.}$ is H_2 partial pressure at the permeate side. In comparison to cubic C-BSCF, hybrid C/H-BSCF had significantly higher proton conductivities, measuring 0.033, 0.029, 0.026, 0.020, and 0.015 S cm^{-1} at various temperatures (650, 600, 550, 500, and 450 °C).

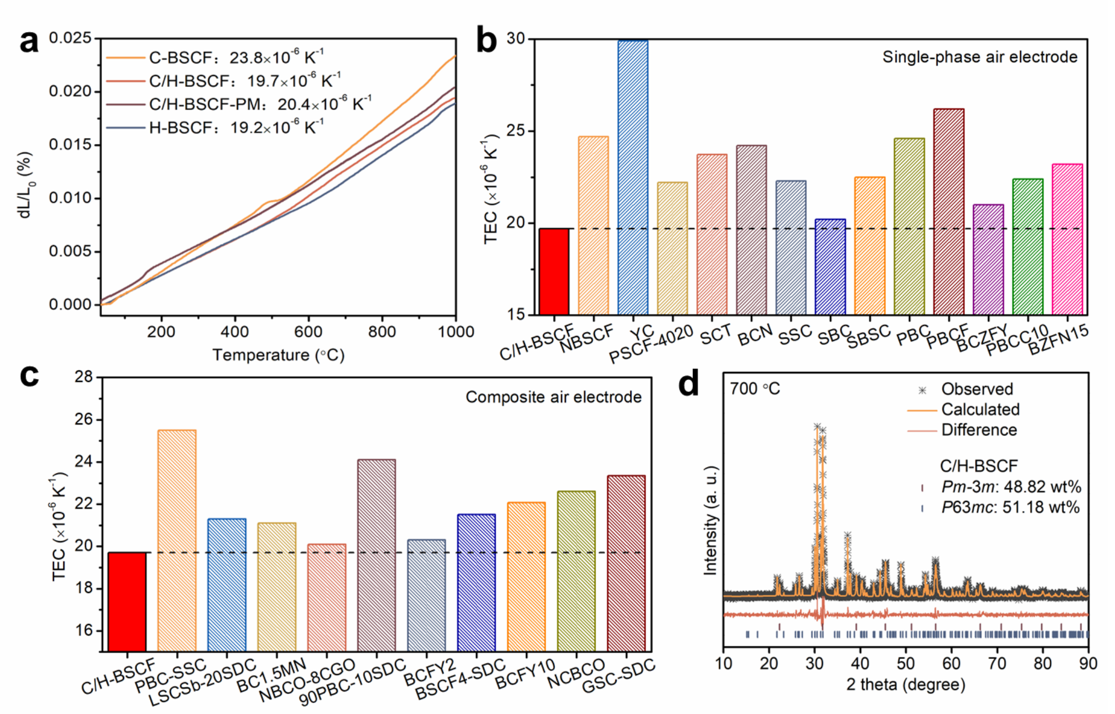

Supplementary Figure 22. a The results of dL/L$_0$ for C-BSCF, C/H-BSCF, C/H-BSCF-PM and H-BSCF from 40 to 1000 °C. TEC comparison of C/H-BSCF electrode with reported advanced (**b**) single-phase and (**c**) composite Co/Fe-based air electrodes [$NdBa_{0.5}Sr_{0.5}Co_{1.5}Fe_{0.5}O_{5+\delta}$ (NBSCF); $Y_{0.96}CoO_{3-\delta}$ (YC); $Pr_{0.6}Sr_{0.4}Fe_{0.8}Co_{0.2}O_{3-\delta}$ (PSCF-4020); $SrCo_{0.95}Ta_{0.05}O_{3-\delta}$ (SCT); $BaCoNbO_{3-\delta}$ (BCN); $Sm_{0.5}Sr_{0.5}CoO_3$ (SSC); $SmBaCo_2O_{5+\delta}$ (SBC); $SmBa_{0.5}Sr_{0.5}Co_2O_{5+\delta}$ (SBSC); $PrBaCo_2O_{5+\delta}$ (PBC); $PrBaCo_{1.5}Fe_{0.5}O_{5+\delta}$ (PBCF); $BaCo_{0.4}Zr_{0.1}Fe_{0.4}Y_{0.1}O_{3-\delta}$ (BCZFY); $PrBa_{0.9}Ca_{0.1}Co_2O_{5+\delta}$ (PBCC10); $BaZr_{0.1}Fe_{0.75}Ni_{0.15}O_{3-\delta}$ (BZFN15); $La_{0.4}Sr_{0.6}Co_{0.9}Sb_{0.1}O_{3-\delta}$-$Ce_{0.8}Sm_{0.2}O_{1.9}$ (LSCSb-20SDC); $Ba_2Co_{1.5}Mo_{0.25}Nb_{0.25}O_{6-\delta}$ (BC1.5MN); $NdBaCo_2O_{5+\delta}$–$Ce_{0.9}Gd_{0.1}O_{1.95}$ (NBCO-8CGO); $BaCe_{0.16}Y_{0.04}Fe_{0.8}O_{3-\delta}$ (BCYF2); $Ba_{0.5}Sr_{0.5}Co_{0.6}Fe_{0.4}O_{3-\delta}$–$Ce_{0.8}Sm_{0.2}O_{1.9}$ (BSCF4-SDC); $BaZr_{0.1}Fe_{0.9}Ni_{0.1}O_{3-\delta}$ (BCFY10); $Gd_{0.1}Ca_{0.1}Ba_{1.8}Co_9O_{14}$ (NCBCO); $Gd_{0.8}Sr_{0.2}CoO_{3-\delta}$-$Ce_{0.8}Gd_{0.2}O_{1.95}$ (GSC-SDC)]. **d** Rietveld refinement XRD profiles of C/H-BSCF hybrid at 700 °C.

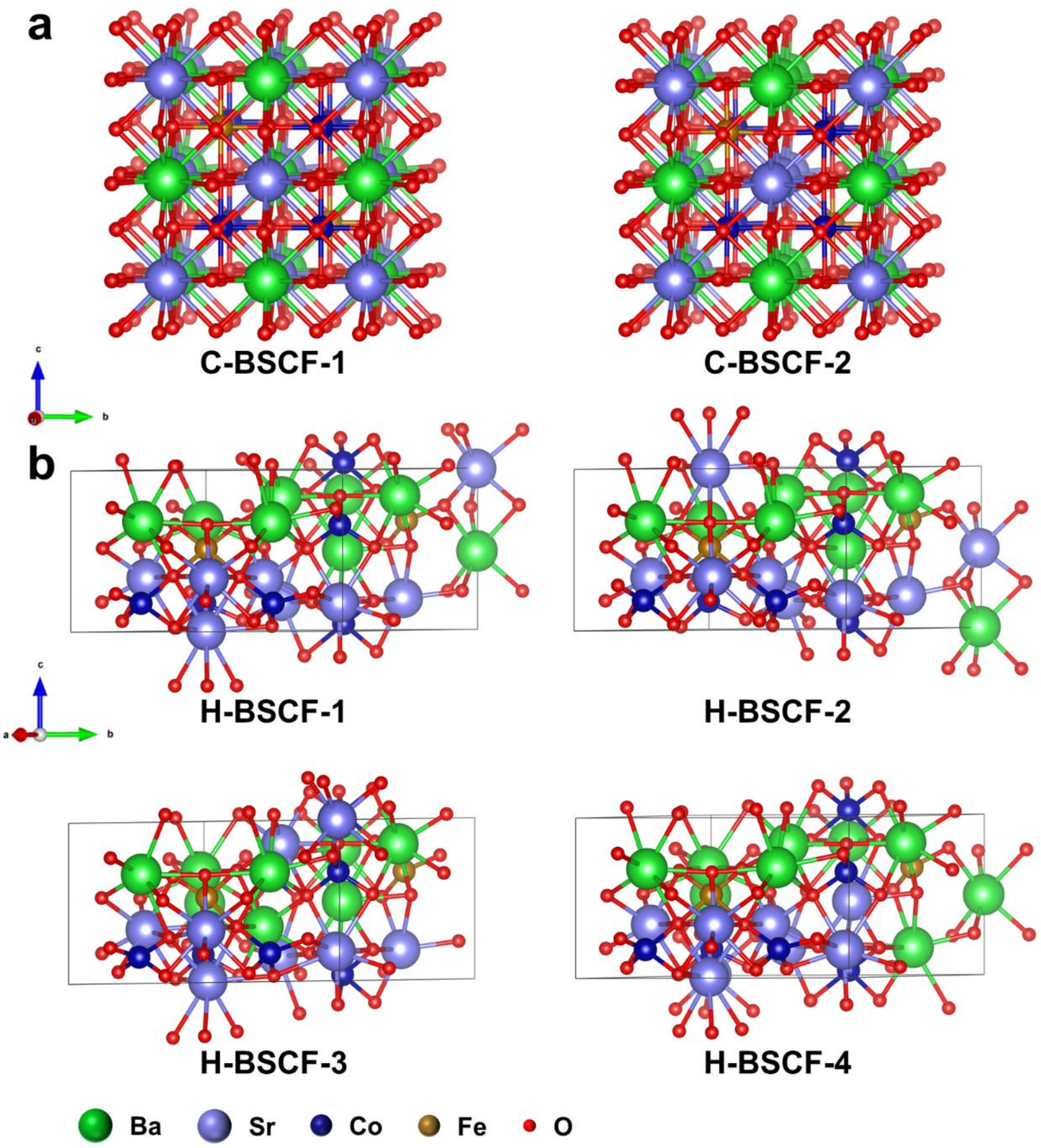

Supplementary Figure 23. Different models of Ba/Sr arrangements for (**a**) cubic C-BSCF, and (**b**) hexagonal H-BSCF.

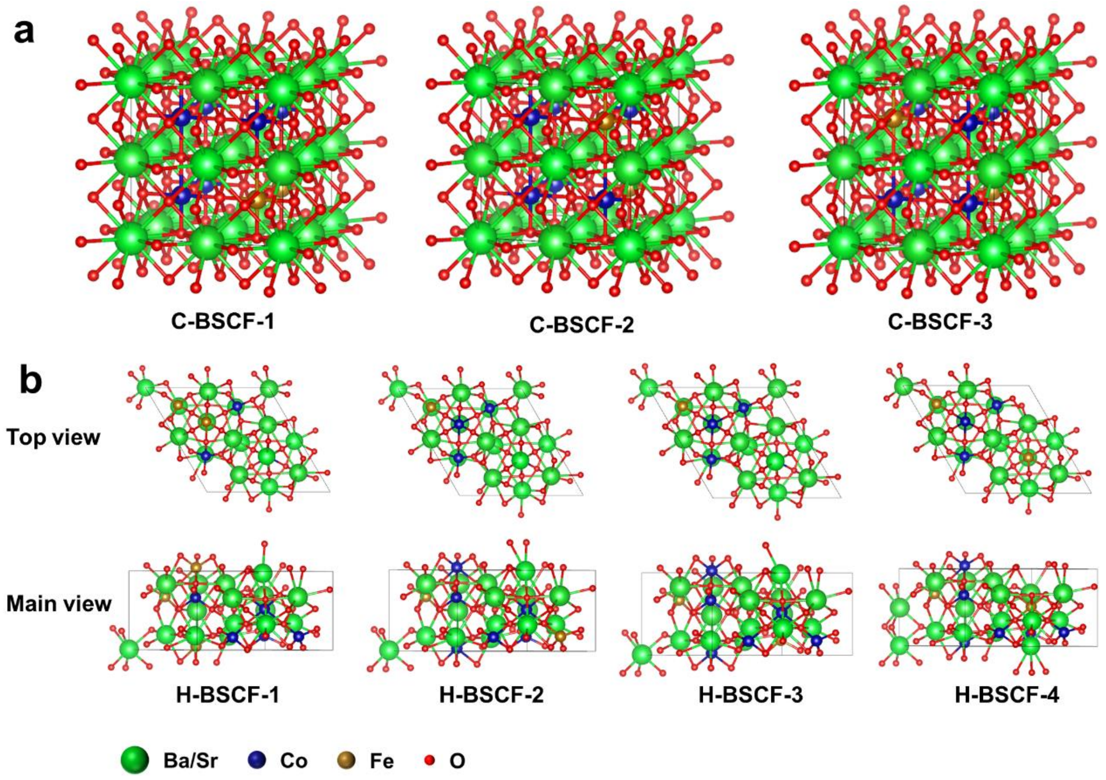

Supplementary Figure 24. Different B-site doped configurations of (**a**) C-BSCF, and (**b**) H-BSCF.

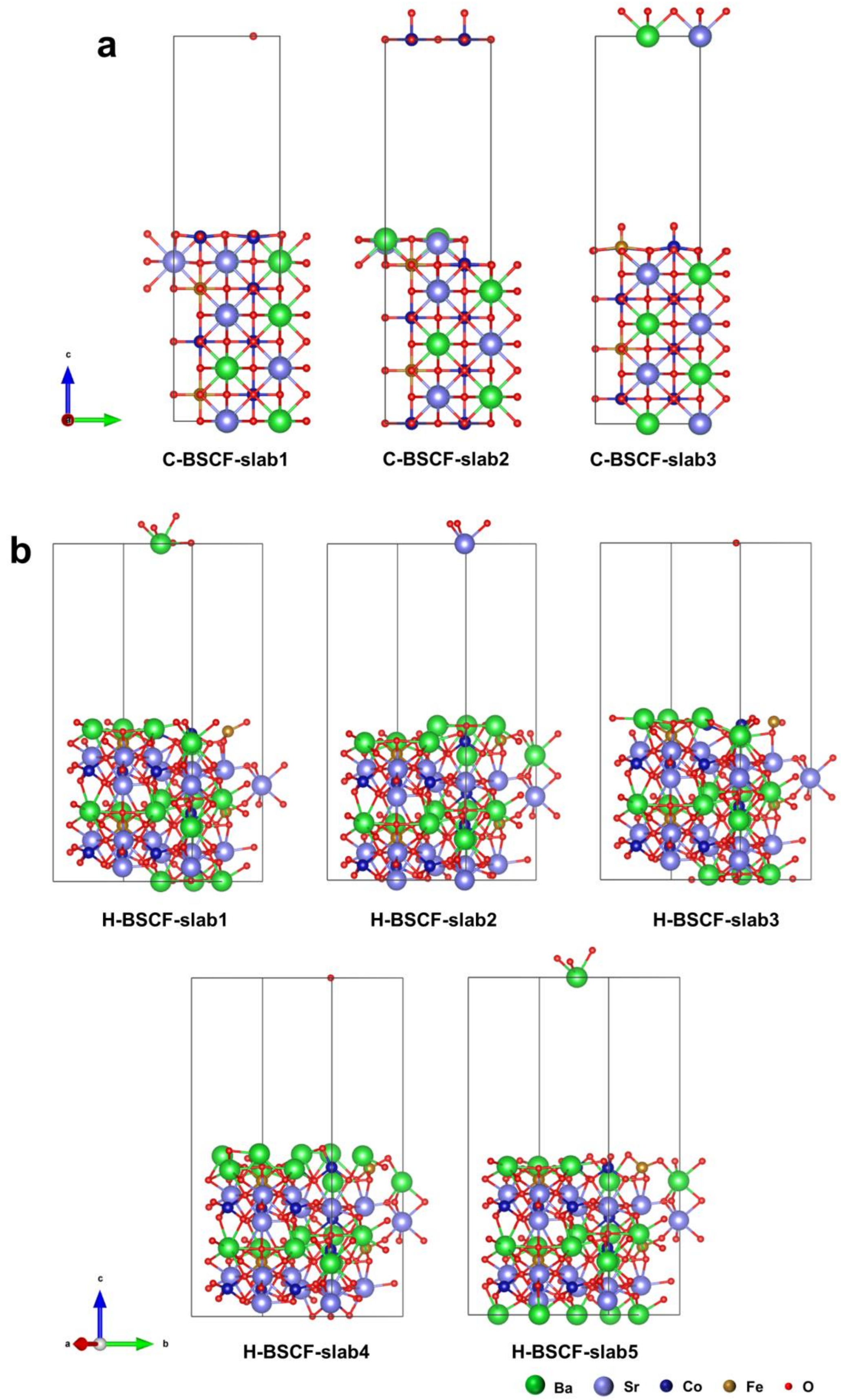

Supplementary Figure 25. Different terminations on the surface of (**a**) C-BSCF, and (**b**) H-BSCF models.

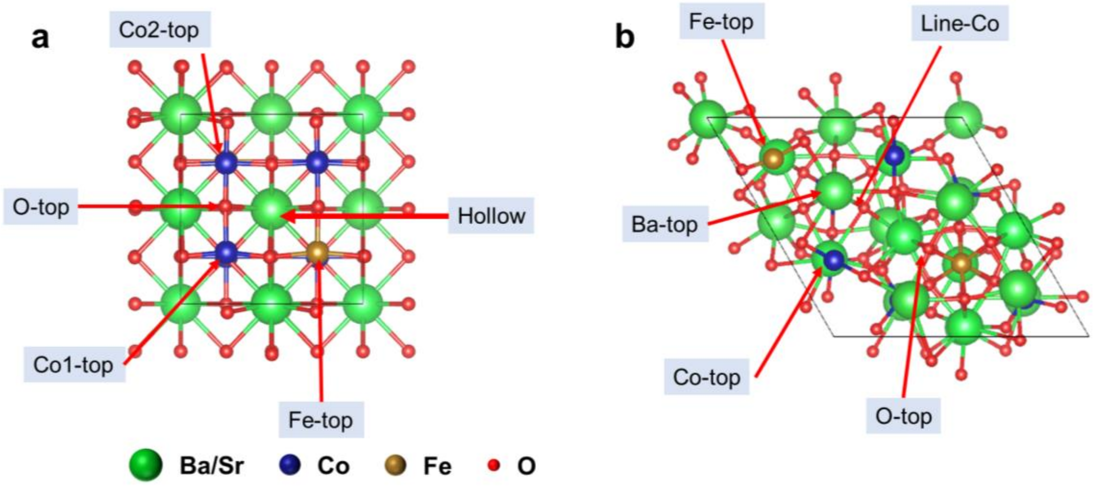

Supplementary Figure 26. Different adsorption sites on the surface of (**a**) C-BSCF, and (**b**) H-BSCF models.

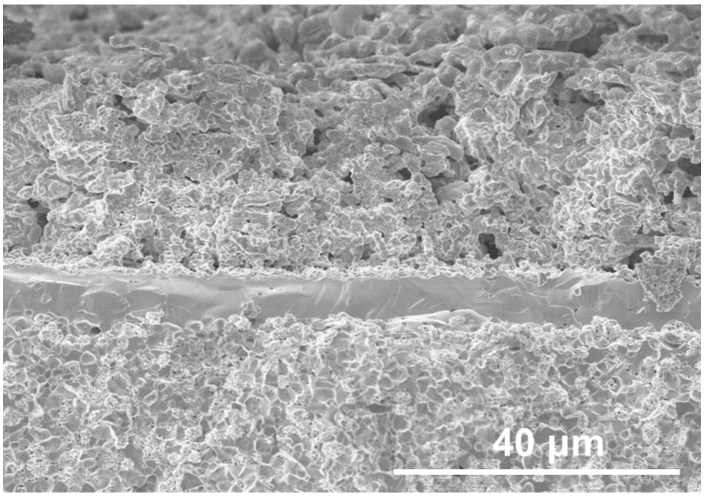

Supplementary Figure 27. SEM image of R-PCEC with C/H-BSCF air electrode.
The highly active and stable C/H-BSCF air electrode was further applied to R-PCECs. Shown in Supplementary Fig. 27 is a cross-sectional view of a test cell, composed of a porous C/H-BSCF air electrode (~ 30 μm), a thin (~ 8 μm) BZCYYb electrolyte, and a Ni-BZCYYb fuel electrode.

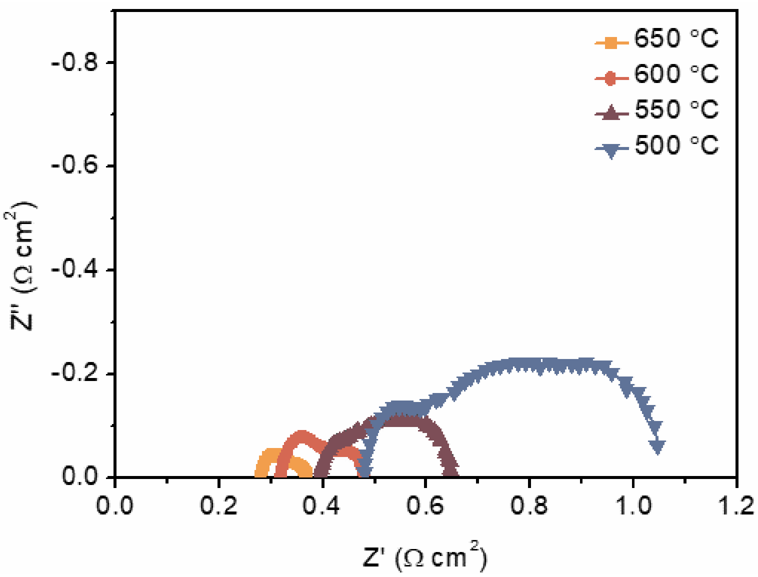

Supplementary Figure 28. EIS of single cell with C/H-BSCF air electrode under open circuit voltage (OCV) condition at 500-650 °C.

Supplementary Fig. 28 shows the typical EIS curves of the cell with the C/H-BSCF air electrode tested at 500-650 °C under open-circuit voltage (OCV) conditions. The cell performance was limited by ohmic resistance (R_o) at temperatures higher than 550 °C, while by R_p at temperatures lower than 550 °C. The cell with C/H-BSCF air electrode has R_p of 0.09, 0.16, 0.25, and 0.56 Ω cm^2 at 650, 600, 550, and 500 °C, respectively.

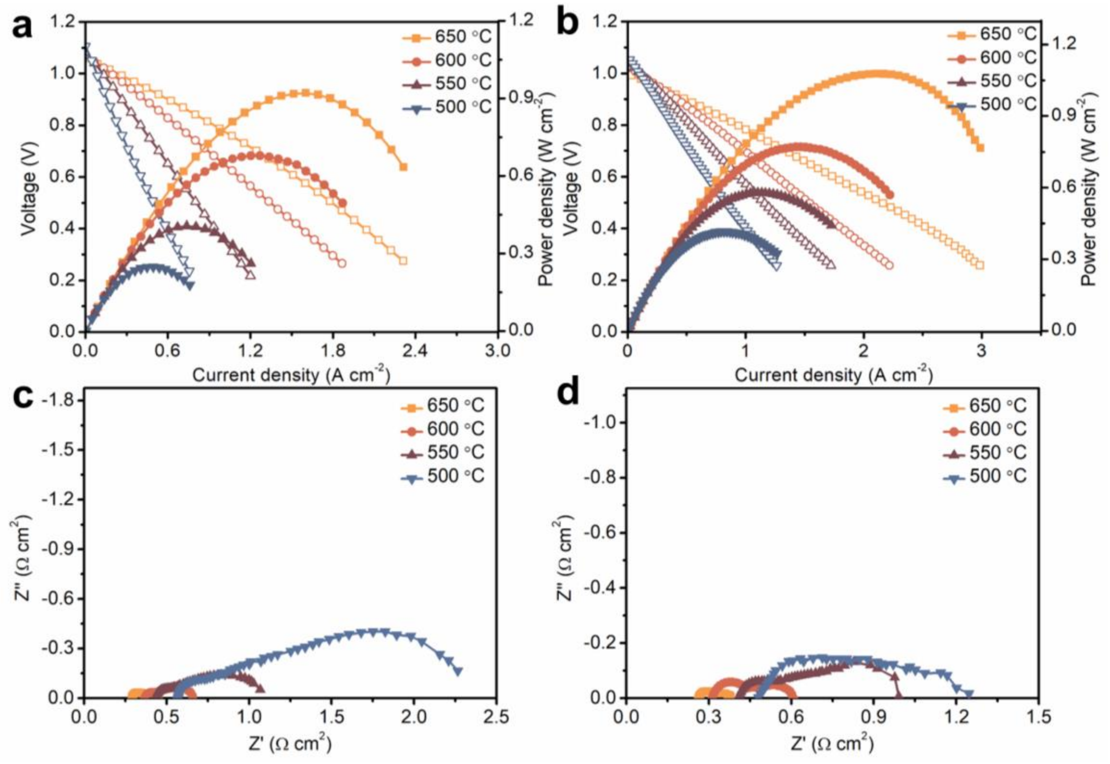

Supplementary Figure 29. *I-V* and *I-P* curves and EIS of single cells with (**a**, **c**) C-BSCF and (**b**, **d**) H-BSCF air electrodes in FC mode at 500-650 °C.

The electrochemical performance of single cells with C-BSCF and H-BSCF air electrodes was extensively studied at 500-650 °C using dry hydrogen on the fuel electrode side and wet air (10% H_2O-air) on the air electrode side for R-PCECs, respectively. *I-V* and *I-P* curves of the C-BSCF cell in FC mode are presented in Supplementary Fig. 29a. The peak power densities (PPDs) of the C-BSCF cell were 0.25, 0.41, 0.68, and 0.92 W cm^{-2} at 500, 550, 600, and 650 °C, respectively. Under the same test conditions, the cell using H-BSCF air electrode obtained higher performance compared to C-BSCF, e.g., PPD of 1.08 W cm^{-2} at 650 °C. This indicates that H-BSCF has higher ORR activity than C-BSCF. In addition, EIS of single cells with C-BSCF and H-BSCF air electrodes were obtained as shown in Supplementary Fig. 29c, d, which further confirmed that due to the excellent catalytic activity of H-BSCF thus exhibited a smaller R_p than the C-BSCF electrode.

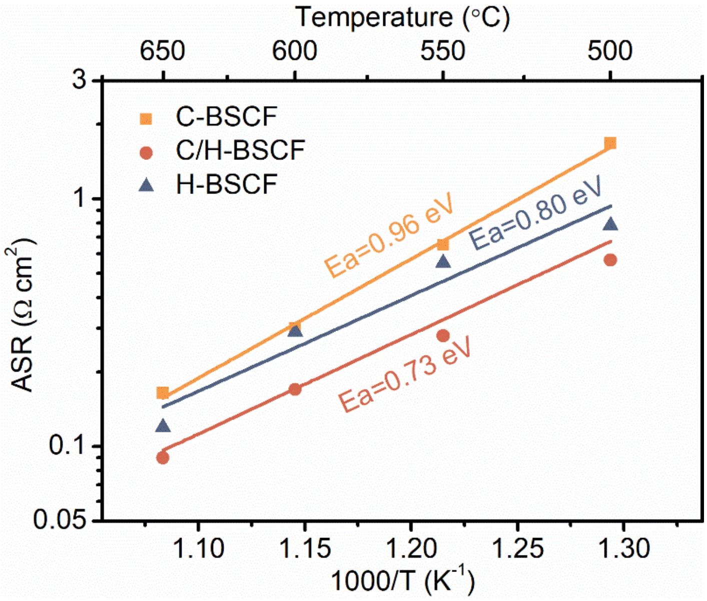

Supplementary Figure 30. Arrhenius plots of the R_p of the C-BSCF, C/H-BSCF and H-BSCF air electrodes in FC mode.

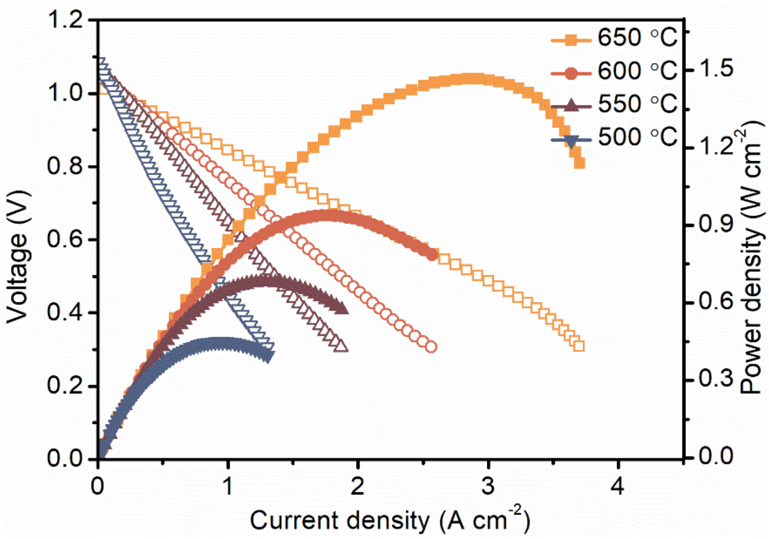

Supplementary Figure 31. *I-V* and *I-P* curves of single cell with C/H-BSCF-PM electrode in FC mode at 500-650 °C.

To further verify the synergistic enhancement of the cubic and hexagonal phases, we prepared C/H-BSCF-PM air electrode by physically mixing C-BSCF and H-BSCF in accordance with the two-phase ratio of hybrid C/H-BSCF. Supplementary Fig. 31 shows the *I-V* and *I-P* curves of single cell with C/H-BSCF-PM electrode in FC mode at 500-650 °C. Despite the catalytic activity of the physically mixed BSCF-1.5-PM oxide is slightly less than that of the hybrid BSCF-1.5, BSCF-1.5-PM still showed good performance as an air electrode for R-PCECs in FC mode with PPDs of 1.47, 0.94, 0.68, and 0.45 W cm^{-2} at 650, 600, 550, and 500 °C, respectively.

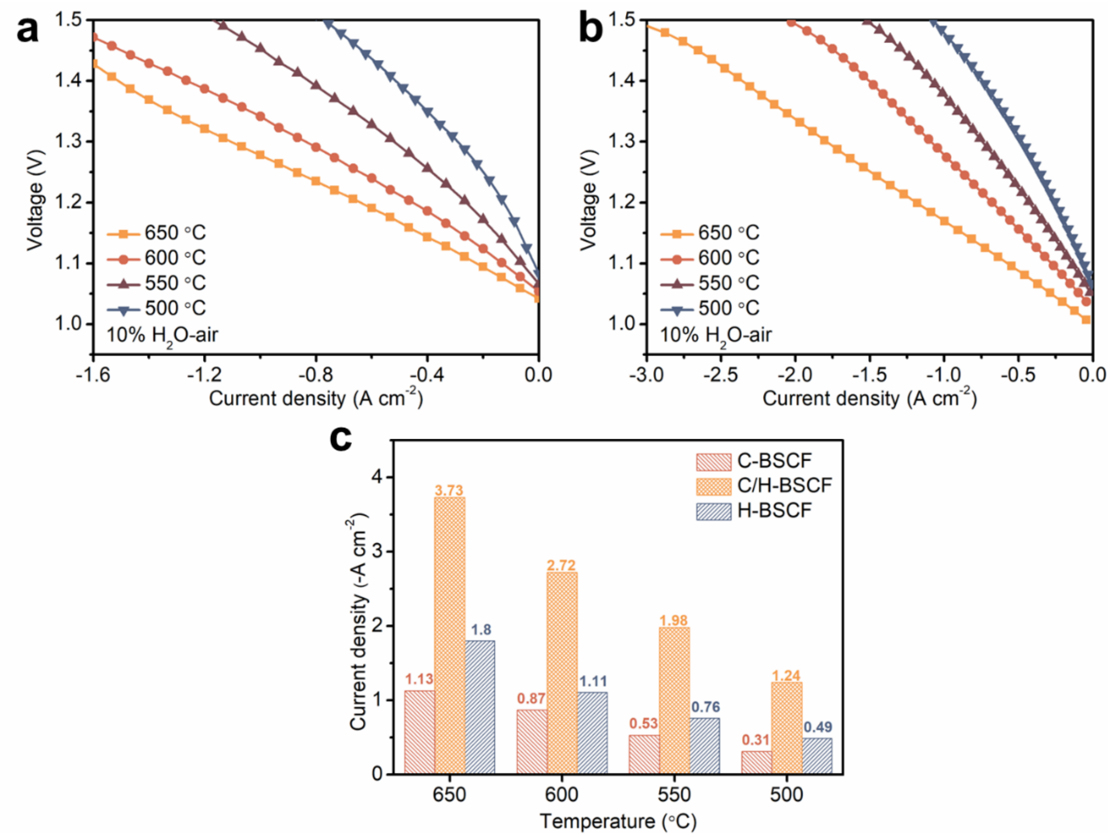

Supplementary Figure 32. *I-V* curves of single cells with (**a**) C-BSCF and (**b**) H-BSCF air electrodes in EC mode at 500-650 °C. **c** Comparison of current densities at different temperatures for single cells with C-BSCF, C/H-BSCF and H-BSCF electrodes.

C-BSCF and H-BSCF as air electrodes for R-PCECs were also tested in electrolysis mode. Supplementary Fig. 32 shows the *I-V* curves of the single cells at 500-650 °C when the fuel electrode was exposed to dry hydrogen and the air electrode supplied with humidified air (10% H_2O-air). The current densities of H-BSCF electrode were -1.80, -1.11, -0.76, and -0.49 A cm^{-2} at 650, 600, 550, and 500 °C, respectively, and the cell voltage was 1.3 V (Supplementary Fig. 32b). Compared with C-BSCF, C/H-BSCF and H-BSCF has excellent electrolytic performance (Supplementary Fig. 32c). For example, at 600 °C, the current density of C/H-BSCF is -2.72 A cm^{-2}, compared with C-BSCF and H-BSCF air electrodes (-0.87 and -1.11 A cm^{-2}), which are improved by 213% and 145%, respectively. Such excellent electrolytic performance of the hybrid C/H-BSCF air electrode is attributed to the synergistic effect of two phases.

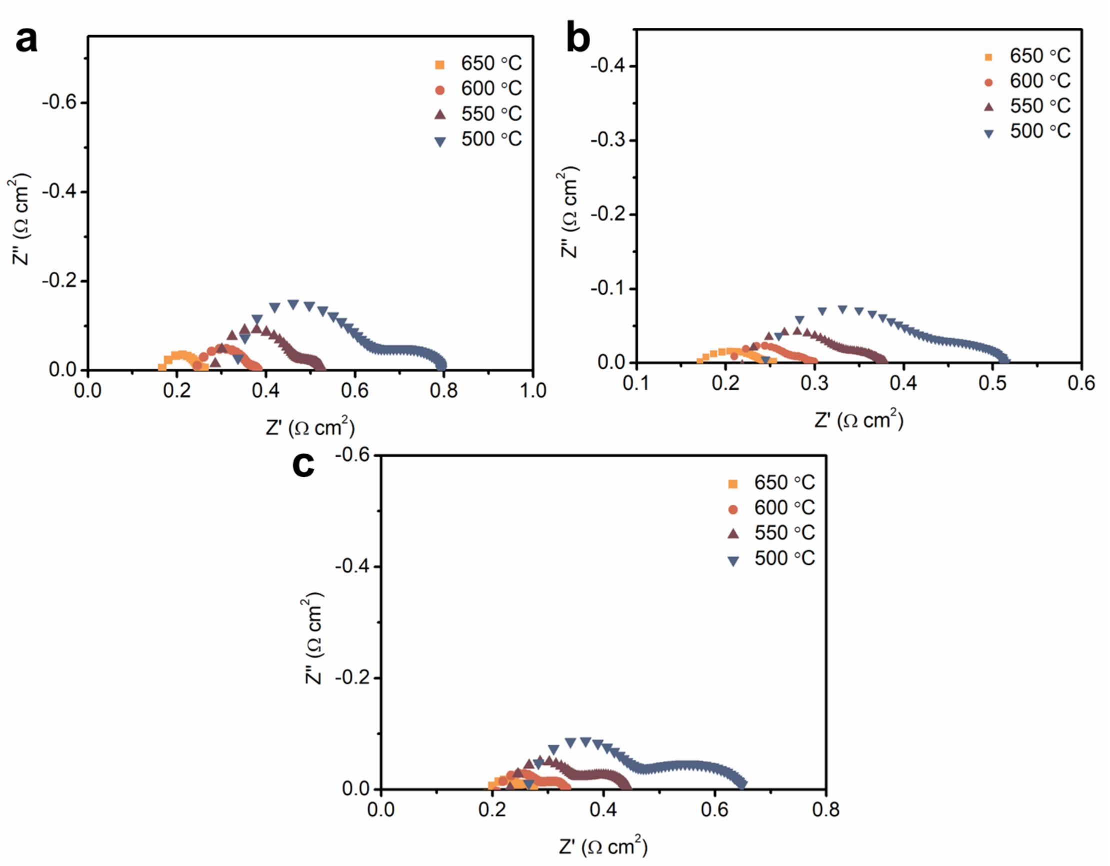

Supplementary Figure 33. EIS of single cells with (**a**) C-BSCF, (**b**) C/H-BSCF and (**c**) H-BSCF air electrodes in EC mode at 500-650 °C.

To reveal the excellent OER activity of the C/H-BSCF air electrode in EC mode, the EIS of the single cell with C/H-BSCF electrode was simultaneously measured under a constant voltage condition of 1.3 V with R_p values of 0.065, 0.110, 0.162, and 0.273 Ω cm^2 at 650, 600, 550, and 500 °C, respectively. At 600 °C, the cells of C-BSCF and H-BSCF electrodes have R_p of 0.152 and 0.135 Ω cm^2, respectively. therefore, the optimal performance obtained for C/H-BSCF electrodes at similar R_o is attributed to their excellent OER activity.

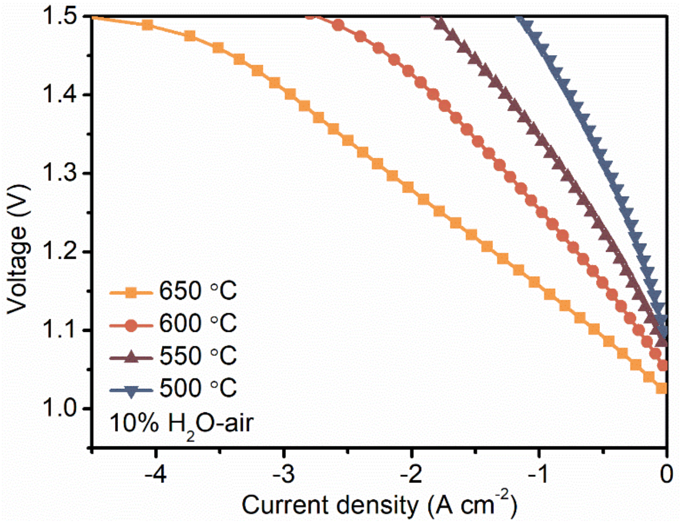

Supplementary Figure 34. *I-V* curves of single cell with C/H-BSCF-PM air electrode in EC mode at 500-650 °C

I-V curves of a single cell with a C/H-BSCF-PM air electrode in EC mode were measured for the evaluation of the OER activity of the electrode with a hybrid of cubic and hexagonal phases. As shown in Supplementary Fig. 34, cell with C/H-BSCF-PM electrode measured high performance of -2.15, -1.23, -0.79, and -0.45 A cm^{-2} at 650, 600, 550, and 500 °C under 10% H_2O-air, respectively, further confirming the result that the dual-phase hybrid oxide has better OER catalytic activity than either phase acting alone.

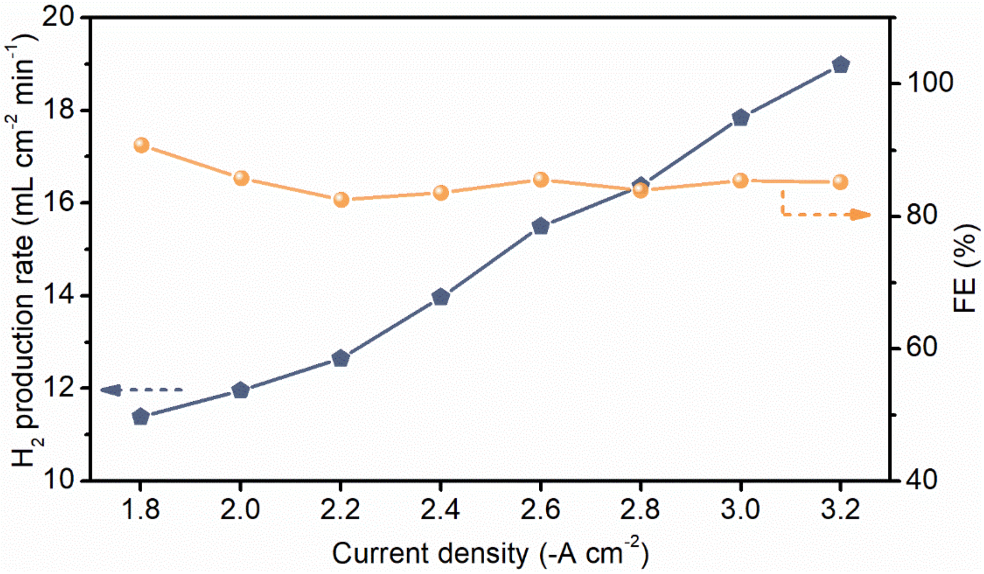

Supplementary Figure 35. Hydrogen production rate and FE of the single cell with C/H-BSCF electrode measured at different current densities at 600 °C under 40% H_2O-air conditions.

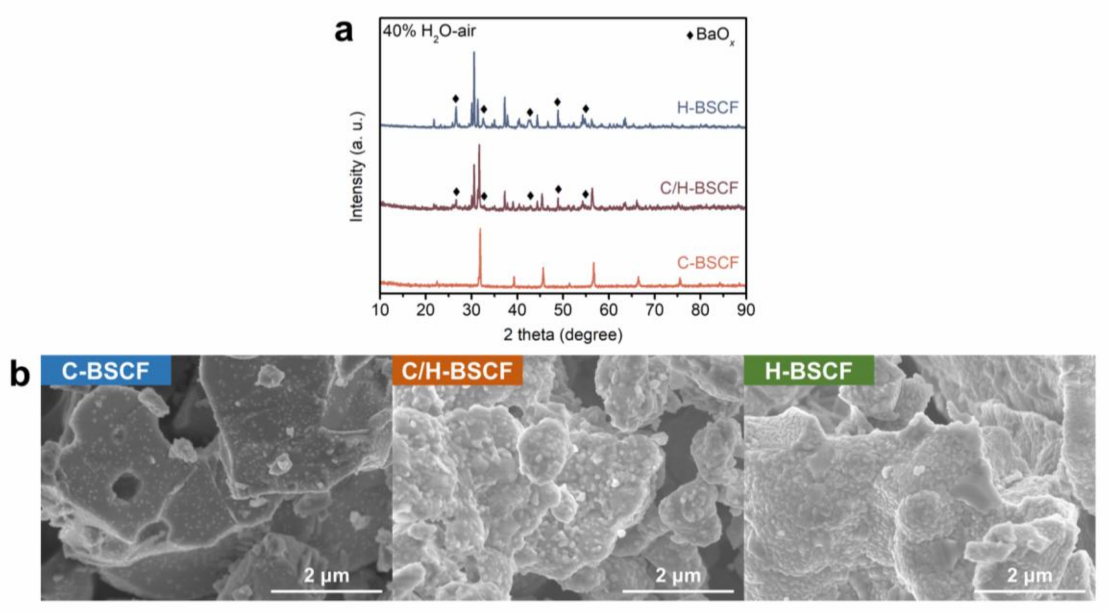

Supplementary Figure 36. a XRD profiles (**b**) and SEM images of the C-BSCF, C/H-BSCF, and H-BSCF powders after treatment at 40% H_2O-air treatment at 600 °C for 10 h.

Supplementary Table 1. XRD refinements information of BSCF-*x* samples (*x* = 1, 1.3, 1.5, 1.7, and 2).

**Sample**	**Crystal parameters**		**Phase proportions (wt.%)**	**Refinement parameters**
C-BSCF	Cubic (*Pm-3m*)	a = 3.9865(3)<br>b = 3.9865(3)<br>c = 3.9865(3)	100	R_{exp} = 5.19<br>R_{wp} = 5.61<br>*GOF* = 1.08
BSCF-1.3	Cubic (*Pm-3m*)	a = 3.9917(0)<br>b = 3.9917(0)<br>c = 3.9917(0)	84.29	R_{exp} = 4.90<br>R_{wp} = 6.84<br>*GOF* = 1.40
	hexagonal (*P63mc*)	a = 11.7077(2)<br>b = 11.7077(2)<br>c = 6.9066(3)	15.71	
C/H-BSCF	Cubic (*Pm-3m*)	a = 3.9967(7)<br>b = 3.9967(7)<br>c = 3.9967(7)	57.26	R_{exp} = 5.44<br>R_{wp} = 5.42<br>*GOF* = 1.00
	hexagonal (*P63mc*)	a = 11.7063(6)<br>b = 11.7063(6)<br>c = 6.9064(0)	42.74	
BSCF-1.7	Cubic (*Pm-3m*)	a = 3.9876(0)<br>b = 3.9876(0)<br>c = 3.9876(0)	45.29	R_{exp} = 5.12<br>R_{wp} = 6.28<br>*GOF* = 1.23
	hexagonal (*P63mc*)	a = 11.7113(0)<br>b = 11.7113(0)<br>c = 6.9050(6)	54.71	
H-BSCF	hexagonal (*P63mc*)	a = 11.7115(7)<br>b = 11.7115(7)<br>c = 6.9075(4)	100	R_{exp} = 5.53<br>R_{wp} = 8.81<br>*GOF* = 1.59

Supplementary Table 2. ASR values of symmetric cells with C-BSCF, H-BSCF and BSCF-*x* (*x* = 1.3, 1.4, 1.5, 1.6, and 1.7) electrodes in the temperature range of 500-700 °C under 3% H_2O-air.

	ASR (Ω cm^2)				
	700 °C	650 °C	600 °C	550 °C	500 °C
C-BSCF	0.126	0.243	0.478	1.093	3.390
BSCF-1.3	0.081	0.186	0.426	1.023	3.407
BSCF-1.4	0.074	0.171	0.387	0.909	3.245
BSCF-1.5	0.050	0.110	0.260	0.680	2.129
BSCF-1.6	0.060	0.143	0.357	0.828	3.311
BSCF-1.7	0.061	0.160	0.390	0.980	3.450
H-BSCF	0.063	0.117	0.343	0.979	4.002

Supplementary Table 3. The fitted values of D_{chem} (cm^2 s^{-1}) and k_{chem} (cm s^{-1}) of C-BSCF, C/H-BSCF, C/H-BSCF-PM and H-BSCF samples at 500-700 °C.

		700 °C	650 °C	600 °C	550 °C	500 °C
C-BSCF	D_{chem}	2.27×10^{-4}	1.33×10^{-4}	4.64×10^{-5}	7.94×10^{-6}	1.42×10^{-6}
	k_{chem}	0.00251	0.00135	4.63×10^{-4}	7.80×10^{-5}	1.46×10^{-5}
C/H-BSCF	D_{chem}	4.88×10^{-4}	3.17×10^{-4}	1.47×10^{-4}	6.00×10^{-5}	1.99×10^{-5}
	k_{chem}	0.00469	0.00298	0.00136	5.48×10^{-4}	1.78×10^{-4}
C/H-BSCF-PM	D_{chem}	1.29×10^{-4}	9.33×10^{-5}	6.21×10^{-5}	3.58×10^{-5}	1.46×10^{-5}
	k_{chem}	0.00125	8.87×10^{-4}	5.93×10^{-4}	3.53×10^{-4}	1.34×10^{-4}
H-BSCF	D_{chem}	4.25×10^{-5}	3.03×10^{-5}	2.03×10^{-5}	1.39×10^{-5}	9.65×10^{-6}
	k_{chem}	4.15×10^{-4}	3.05×10^{-4}	2.03×10^{-4}	1.39×10^{-4}	9.67×10^{-5}

Supplementary Table 4. Energies of different models of Ba/Sr arrangements for C-BSCF and H-BSCF.

Configuration names	Total energies (eV)
C-BSCF-1	-242.423
C-BSCF-2	-242.233
H-BSCF-1	-330.000
H-BSCF-2	-329.907
H-BSCF-3	-329.871
H-BSCF-4	-329.706

Supplementary Table 5. Energies of different B-site doped configurations of C-BSCF and H-BSCF.

Configuration names	Total energies (eV)
C-BSCF-1	-242.236
C-BSCF-2	-242.955
C-BSCF-3	-243.068
H-BSCF-1	-329.998
H-BSCF-2	-329.745
H-BSCF-3	-329.729
H-BSCF-4	-330.257

Supplementary Table 6. Total energies and $E_{surface}$ of Different terminations on the surface for C-BSCF and H-BSCF models.

Configuration names	Total energies (eV)	$E_{surface}$ (eV)
C-BSCF-slab1	-491.558	-0.0549
C-BSCF-slab2	-491.533	-0.0547
C-BSCF-slab3	-474.561	0.0841
H-BSCF-slab1	-667.552	-0.0314
H-BSCF-slab2	-666.077	-0.0253
H-BSCF-slab3	-658.005	0.0083
H-BSCF-slab4	-654.677	0.0222
H-BSCF-slab5	-659.700	0.0012

Supplementary Table 7. Adsorption energies of different sites on the surfaces of C-BSCF and H-BSCF models.

	Adsorption sites	Total energies (eV)
C-BSCF	Co1-top	-327.561
	Co2-top	-327.605
	Fe-top	-327.766
	Hollow	-327.072
	O-top	-327.098
H-BSCF	Ba-top	-491.811
	Co-top	-491.253
	Fe-top	-492.132
	Line-Co	-492.035
	O-top	-492.206

Supplementary Table 8. Frequencies of the structures of the C-BSCF and H-BSCF reaction processes.

		**Frequencies** (cm^{-1})						
		$*H_2O$	*OH+*H	*OH	*OH(TS)	*O+*H	*O	$*O_2$
C-BSCF	1f	3712.524	3715.298	3698.838	1534.093	3431.05	539.747	1536.215
	2f	3306.291	3370.782	691.5385	893.0461	875.599	143.2482	112.6365
	3f	1554.014	946.7179	516.0957	630.6273	700.9091	121.4698	66.12432
	4f	725.7877	699.0307	151.0977	511.1154	368.7323		50.41883
	5f	579.6506	534.6541	132.7959	170.809	188.3148		24.84868
	6f	309.7386	483.7285	19.55591	588.3934	179.2097		13.80394
	7f	280.0206	228.1486					
	8f	137.8007	172.1399					
	9f	82.68794	121.7279					
H-BSCF	1f	3768.161	3773.422	3762.472	1825.158	3142.869	601.7816	879.6629
	2f	3644.396	2969.247	522.7825	1188.135	978.4043	235.1142	443.9459
	3f	1545.859	1029.351	440.1786	668.2542	698.8283	165.1762	428.4271
	4f	452.0684	815.571	251.2754	365.8682	535.0296		185.6172
	5f	377.3766	580.0342	106.7067	154.737	267.771		165.6534
	6f	307.6515	459.9621	70.92847	790.9388	213.8166		70.56255
	7f	173.7961	246.218					
	8f	70.90124	138.1902					
	9f	41.73783	80.61461					

Supplementary Table 9. ZPE and TΔS of the structures of the C-BSCF and H-BSCF reaction processes.

	Reaction steps	ZPE	TΔS
C-BSCF	$*H_2O$	0.662606	0.133428
	*OH+*H	0.636799	0.117254
	*OH	0.322974	0.083943
	*OH(TS)	0.231832	0.046634
	*O+*H	0.356072	0.079106
	*O	0.049871	0.082707
	$*O_2$	0.111837	0.221142
H-BSCF	$*H_2O$	0.643598	0.072555
	*OH+*H	0.625664	0.12906
	*OH	0.31953	0.13701
	*OH(TS)	0.260501	0.052986
	*O+*H	0.361832	0.059495
	*O	0.062121	0.061685
	$*O_2$	0.134763	0.085047

References

1. Zhong, F. et al. Geometric structure distribution and oxidation state demand of cations in spinel $Ni_xFe_{1-x}Co_2O_4$ composite cathodes for solid oxide fuel cells. *Chem. Eng. J.* **425**, 131822 (2021).
2. Guan, D. et al. Utilizing ion leaching effects for achieving high oxygen-evolving performance on hybrid nanocomposite with self-optimized behaviors. *Nat. Commun.* **11**, 3376 (2020).
3. Kim, J. H. et al. Self-assembled nano-composite perovskites as highly efficient and robust hybrid cathodes for solid oxide fuel cells. *J. Mater. Chem. A* **10**, 2496-2508 (2022).
4. Pei, K. et al. Constructing an active and stable oxygen electrode surface for reversible protonic ceramic electrochemical cells. *Appl. Catal. B: Environ.* **330**, 122601 (2023).
5. Kuai, X. et al. Boosting the activity of $BaCo_{0.4}Fe_{0.4}Zr_{0.1}Y_{0.1}O_{3-\delta}$ perovskite for oxygen reduction reactions at low-to-intermediate temperatures through tuning B-site cation deficiency. *Adv. Energy Mater.* **9**, 1902384 (2019).
6. Liu, Z. et al. Robust bifunctional phosphorus-doped perovskite oxygen electrode for reversible proton ceramic electrochemical cells. *Chem. Eng. J.* **450**, 137787 (2022).
7. Zhu, Y. et al. Oxygen activation on Ba-containing perovskite materials. *Sci. Adv.* **8**, eabn4072 (2022).
